# Unique phylogenies and metabolic adaptations of novel lineage III and comammox *Nitrospira* species from deep-sea sediments

**DOI:** 10.1093/ismeco/ycag003

**Published:** 2026-01-09

**Authors:** Guohao Chen, Hongmei Jing, Bolin Liu, Jiawei Zhang, Yafei Ou, Wenxiao Liu, Xinru Tian, Ran Wang, Jinlin Yan, Tieqiang Mao, Sai Yang, Yanling Zheng, Lijun Hou, Hongpo Dong

**Affiliations:** State Key Laboratory of Estuarine and Coastal Research, Yangtze Delta Estuarine Wetland Ecosystem Observation and Research Station, East China Normal University, Shanghai 200241, China; CAS Key Laboratory for Experimental Study Under Deep-Sea Extreme Conditions, Institute of Deep-Sea Science and Engineering, Chinese Academy of Sciences, Sanya 572000, China; State Key Laboratory of Estuarine and Coastal Research, Yangtze Delta Estuarine Wetland Ecosystem Observation and Research Station, East China Normal University, Shanghai 200241, China; State Key Laboratory of Estuarine and Coastal Research, Yangtze Delta Estuarine Wetland Ecosystem Observation and Research Station, East China Normal University, Shanghai 200241, China; State Key Laboratory of Estuarine and Coastal Research, Yangtze Delta Estuarine Wetland Ecosystem Observation and Research Station, East China Normal University, Shanghai 200241, China; State Key Laboratory of Estuarine and Coastal Research, Yangtze Delta Estuarine Wetland Ecosystem Observation and Research Station, East China Normal University, Shanghai 200241, China; State Key Laboratory of Estuarine and Coastal Research, Yangtze Delta Estuarine Wetland Ecosystem Observation and Research Station, East China Normal University, Shanghai 200241, China; State Key Laboratory of Estuarine and Coastal Research, Yangtze Delta Estuarine Wetland Ecosystem Observation and Research Station, East China Normal University, Shanghai 200241, China; State Key Laboratory of Estuarine and Coastal Research, Yangtze Delta Estuarine Wetland Ecosystem Observation and Research Station, East China Normal University, Shanghai 200241, China; State Key Laboratory of Estuarine and Coastal Research, Yangtze Delta Estuarine Wetland Ecosystem Observation and Research Station, East China Normal University, Shanghai 200241, China; State Key Laboratory of Estuarine and Coastal Research, Yangtze Delta Estuarine Wetland Ecosystem Observation and Research Station, East China Normal University, Shanghai 200241, China; Key Laboratory of Geographic Information Science of the Ministry of Education, School of Geographic Sciences, East China Normal University, Shanghai 200241, China; State Key Laboratory of Estuarine and Coastal Research, Yangtze Delta Estuarine Wetland Ecosystem Observation and Research Station, East China Normal University, Shanghai 200241, China; State Key Laboratory of Estuarine and Coastal Research, Yangtze Delta Estuarine Wetland Ecosystem Observation and Research Station, East China Normal University, Shanghai 200241, China

**Keywords:** *Nitrospira* lineages, comammox, deep sea, sediments, metagenome-assembled genomes, geographical distribution

## Abstract

The genus *Nitrospira*, which includes canonical nitrite-oxidizing bacteria (NOB) and species capable of complete ammonia oxidation (comammox), plays an important role in the global biogeochemical nitrogen cycle. Typically, lineage IV *Nitrospira* predominate in marine environments, and other lineages are thought to be less abundant and remain poorly characterized in oceanic systems. Here, we recovered five novel metagenome-assembled genomes (MAGs) affiliated with *Nitrospira* lineage II–IV from deep-sea sediments. Notably, two of these MAGs represent members of lineage III and comammox *Nitrospira*, respectively, suggesting the presence of previously uncharacterized lineages in the deep sea. Phylogenetic and gene locus analyses indicated that deep-sea lineage III and comammox *Nitrospira* form distinct evolutionary clades that diverge from their terrestrial and coastal relatives, and we therefore designate these two marine-derived groups as “lineage III clade B” and “comammox clade A4”, respectively. Comparative read recruitment analyses revealed that these lineages exhibit potential pan-oceanic distribution in deep-sea sediments and waters, albeit at very low abundances. Furthermore, the identification of genes encoding *amtB*-type ammonium transporters (*amtB*), the ABC-type glycerol-3-phosphate transport system (*ugpABCE*), a multi-subunit Na^+^/H^+^ antiporter (*mnh*), and betaine transporters (*BetT*, *opuABC*) suggests that these newly discovered *Nitrospira* species possess adaptive capabilities to thrive in oligotrophic and saline marine environments. These findings provide novel insights into the occurrence, metabolic features, and adaptation strategies of lineage III and comammox *Nitrospira*, expand our understanding of *Nitrospira* diversity in the deep sea, and offer valuable perspectives on the evolutionary history of various *Nitrospira* lineages.

## Graphical Abstract

**Graphical Abstract:**
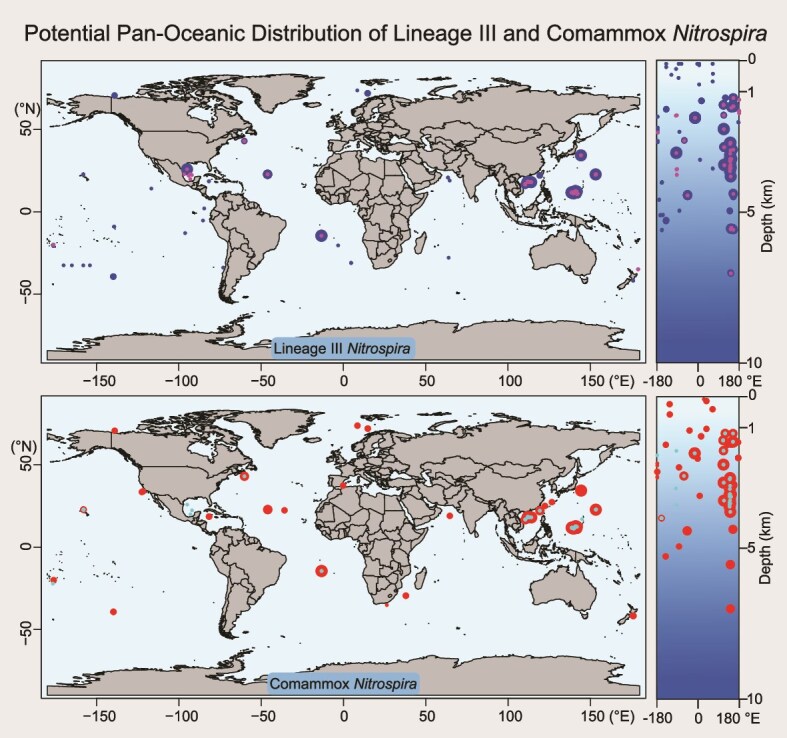

## Introduction

The ocean represents the largest reservoir of reactive nitrogen on Earth, with this nutrient often acting as a co-limiting factor for marine organisms, thereby driving ocean productivity and sustaining complex ecosystems [1]. Nitrification, a critical biological process oxidizing ammonia to nitrate via nitrite, bridges the oxidized and reduced pools of inorganic nitrogen, and plays a crucial role in the global marine nitrogen cycle. Until 2015, nitrification was generally considered to be a two-step process mediated by canonical ammonia oxidizers and nitrite-oxidizing bacteria (NOB) [2]. However, the paradigm-shifting discovery of complete ammonia oxidizer (comammox) within lineage II *Nitrospira* revealed microorganisms capable of oxidizing ammonia to nitrate entirely within a single cell [3, 4]. As the most phylogenetically diverse genus of NOB, *Nitrospira* contain at least six sub-lineages, exhibiting remarkable metabolic versatility and a cosmopolitan distribution across natural and engineered ecosystems, including marine environments, lakes, groundwater, soils, drinking water systems and waste-water treatment plants, and activated sludge [5], thereby serving as key participants in global nitrogen cycling. In the latest release of GTDB (r226) [6], *Nitrospira* are now reclassified into different genera within the order *Nitrospirales*, reflecting broad phylogenetic and functional diversity based on comparative genome analysis [7]. Recent studies have further demonstrated that NOB, including *Nitrospira*, play an important role in dark ocean chemoautotrophy, highlighting the underestimated influence of nitrite oxidation on the marine carbon cycle [8, 9].

To date, members of lineage IV *Nitrospira*, exemplified by the cultured strain *Nitrospira marina* Nb-295, have been predominantly identified in open-ocean environments [5, 10, 11]. Comammox *Nitrospira* have been frequently reported in terrestrial ecosystems [12–14], but not in the open ocean. It is widely recognized that ammonia-to-nitrate conversion in the open ocean occurs exclusively via a partnership between canonical ammonia oxidizers and NOB [8, 15]. Nonetheless, comammox *Nitrospira* have been frequently detected in coastal ecosystems, including mangrove wetlands [13], estuarine salt marshes [16], and adjacent seas [17, 18], suggesting previously unrecognized halotolerance potential. These findings raise critical questions about the ecological distribution of *Nitrospira* and their physiological diversity in nitrogen cycling within deep-sea habitats.

Canonical *Nitrospira* exhibit a high affinity for nitrite (*K_m_* = ~9–27 μM), indicating their ability to inhabit oligotrophic environments with low nitrite concentrations [19]. Beyond nitrite oxidation, certain *Nitrospira* strains possess chemoautotrophic growth capabilities by oxidizing hydrogen and formate, with oxygen or nitrate as terminal electron acceptors [20, 21]. Many *Nitrospira* species can hydrolyze urea and cyanate to ammonia, facilitating reciprocal interactions with ammonia oxidizers [5]. Moreover, Sakoula et al. [22] enriched a comammox species, *Candidatus Nitrospira kreftii*, which harbors genes encoding a Na^+^-translocating N-ATPase (*atp*) and a sodium-pumping complex I (*nqr*), exhibiting a degree of salt tolerance that enables its survival in brackish ecosystems [23, 24]. The remarkable metabolic diversity and environmental adaptability of *Nitrospira* underpin their widespread distribution across various habitats [15]. Yet, our understanding of how these diverse metabolic strategies influence the ecological distribution of other marine *Nitrospira* lineages and their evolutionary relationships remains limited.

To fill the knowledge gap, we employed a metagenomic approach to recover five novel metagenome-assembled genomes (MAGs) from deep-sea sediments. Our analysis revealed two MAGs affiliated with lineage III and comammox *Nitrospira* that have not been reported in the deep sea. By establishing the largest *Nitrospira* genomes database to date for comparative analyses, we uncovered the metabolic versatility and niche adaptation mechanisms of these newly recovered lineage III and comammox *Nitrospira* MAGs in deep-sea environments. These findings expand the known diversity of the *Nitrospira* genus in the deep ocean, and provide metagenomic evidence for the presence of lineage III and comammox *Nitrospira* in deep-sea habitats.

## Materials and methods

### Sample collection and sequencing

In this study, we collected 17 sediment samples from a deep-sea mount region (abbreviated as DPSM) in the western Pacific Ocean at depths ranging from 2083 to 3456 m using a push corer deployed on the manned submergence vehicle *Deep-Sea Warrior* (Supplementary Fig. S1; Supplementary Table S1). Hydrographic parameters were measured *in situ* using the submersible, and samples were immediately stored at −80°C for further analysis, as previously described [25]. Genomic DNA was extracted from 5–10 g of sediments using a DNA extraction kit (MoBio Laboratories, Carlsbad, USA), and DNA quality was assessed using a Qubit 2.0 fluorometer and a NanoDrop One spectrophotometer (Thermo Fisher Scientific, Waltham, USA). Metagenomic sequencing was performed on a NovaSeq 6000 platform (Illumina, CA, USA) at Novogene Co., Ltd. (www.novogene.com), generating ~15 Gbp of raw reads per sample.

### Metagenomic assembly and genome reconstruction

Raw reads were quality trimmed and adapter sequences were removed using Trimmomatic [26] (v0.39). The read pairs were co-assembled using MEGAHIT [27] (v1.2.9) with the parameter: –presets meta-large. Contigs with length ≥ 2000 bp were binned using MetaBAT2 [28] (v2.15) with eight sensitivity parameter combinations (−maxP 60 or 95, −minS 60 or 95, −maxEdges 200 or 500). Resulting bins were subsequently summarized and filtered using DAS_Tool [29] (v1.1.1). The completeness and contamination of the recovered MAGs were assessed using CheckM [30] (v1.1.3), and their taxonomic assignments were determined using GTDB-Tk [6] (v2.4.1) based on the Genome Taxonomy Database (release 226).

The resulting MAGs then underwent a quality control and refinement pipeline to improve their accuracy and reliability. Briefly, contigs were annotated using CAT [31] (v5.2.3), and those assigned to non-target phyla were systematically removed. Duplicate contigs were curated manually through analysis of single-copy marker genes generated by CheckM [30] (v1.1.3). Outlier contigs were excluded based on variability of GC content, discrepancies of sequencing coverage, and deviations of tetranucleotide frequency using RefineM [32] (v0.1.2). Potential misassigned contigs within MAGs were checked and manually re-binned using Emergent Self-Organizing Maps (ESOM) [33] (v2.0.4). A total of five high- and medium-quality *Nitrospira*-like MAGs meeting stringent quality thresholds (>74% completeness and < 4% contamination) were recovered (Supplementary Table S2) [34]. Pairwise average nucleotide identity (ANI) values among these MAGs (Supplementary Table S3) were calculated using FastANI [35] (v1.32).

### Phylogenomic analysis of concatenated marker proteins

A total of 299 representative genomes, including 279 uncultured *Nitrospirae* genomes from the NCBI and IMG databases, 14 cultured *Nitrospira* genomes, three lineage III *Nitrospira* MAGs recovered from coastal mangrove sediments, and three *Nitrospinae* genomes (outgroup), were selected to infer the phylogeny of the five recovered *Nitrospira*-like MAGs (Supplementary Table S2). Briefly, the reference genomes (>70% completeness and <5% contamination) were selected and analyzed using QUAST [36] (v5.0.2) to assess genomic features such as GC content, genome size, and contig metrics. A concatenated set of 120 bacterial marker proteins was extracted using GTDB-Tk [6] (v2.4.1), and a maximum-likelihood (ML) phylogenetic tree was constructed using IQ-TREE [37] (v1.6.12) with the LG + F + R10 model and 1000 ultrafast bootstrap replicates. The resulting phylogenomic tree was visualized using ITOL [38] (v7.1). Moreover, relative evolutionary divergence (RED) values were estimated based on the phylogenomic tree as previously described [39].

### Phylogenetic analysis of key marker genes

*Nitrospira* lineages have historically been classified based on 16S rRNA or nitrite oxidoreductase beta subunit (*nxrB*) genes [40, 41]. Here, two MAGs—representing lineage III (DS64) and clade A comammox *Nitrospira* (DS176)—were recovered from deep-sea sediments. Phylogenetic analyses were conducted on key marker genes from these genomes, including *nxrB* and the genes encoding ammonia monooxygenase (AMO) alpha (*amoA*) and beta (*amoB*) subunits, as well as hydroxylamine dehydrogenase (HAO) alpha (*haoA*) and beta (*haoB*) subunits. Sequences of these marker genes were identified from deep-sea MAGs and metagenomes using Prodigal [42] (v2.6.3) and BLASTn [43] (v2.9.0), and reference sequences were retrieved from other *Nitrospira*-like genomes or the NCBI database. The combined set of marker gene sequences was then aligned using MAFFT [44] (v7.402) and trimmed with BMGE [45] (v1.12). ML phylogenetic trees were constructed using IQ-TREE [37] (v1.6.12) with 1000 ultrafast bootstrap replicates, and subsequently visualized using iTOL [38] (v7.1). Detailed information of sequence phylogenies for various genes is provided in the Supplementary Methods.

### Distribution and abundance analysis of MAGs DS64 and DS176 in the global ocean

The distribution and abundance of the MAGs DS64 and DS176 (affiliated with lineage III and comammox *Nitrospira*, respectively) in the global ocean were estimated via read recruitment. A total of 487 marine metagenomes, comprising 17 from this study and 470 from the NCBI Sequence Read Archive, were analyzed. These metagenomes were sourced from various marine environments across global oceans, including epipelagic zone waters (150), mesopelagic zone waters (40), hydrothermal vents (13), deep-sea water (64), and deep-sea sediments (220), with sampling depths ranging from 5 to 9161 m (Supplementary Tables S4 and S5). Indices for the two MAGs sequences were built using BWA-index, and the reads were mapped to these indices using BWA-MEM [46] (v0.7.17). The mapped reads were then filtered using CoverM [47] (v0.3.1) with the parameters: –min-read-percent-identity 0.98, –min-read-aligned-percent 0.9, and –min-read-aligned-length 100. Filtered reads with counts ≥20 were used to calculate the Reads Per Kilobase per Million mapped reads (RPKM) [mapped reads / (metagenomic reads × genome length) × 10^6^]. Abundance data were visualized as solid circles on a world map using the *maps* package (v3.4.2.1) in R (v3.5.3).

Relative abundance and distribution of marker genes (*nxrB* for lineage III; *amoA* and *amoB* for comammox *Nitrospira* genomes) were also determined through comparative read recruitment. For lineage III *nxrB*, a database with nearly full-length *nxrB* genes (>1000 bp) from *Nitrospina* and *Nitrospira* lineages I-VI was indexed and aligned with the marine metagenomic datasets. The resulting reads were filtered with CoverM [47] (v0.3.1) (parameters: –min-read-percent-identity 0.95 –min-read-aligned-percent 0.5 –min-read-aligned-length 75), and reads mapped to *Nitrospira* lineage III *nxrB* sequences were quantified. To reduce false positives during recruitment, the short reads were assembled using SPAdes [48] (v3.15.2) and identified through phylogenetic analysis (Supplementary Methods). Filtered reads with counts ≥2 were used to calculate the reads Count Per Million (CPM) [(number of reads mapped in metagenomes / total read number) × 10^6^], and plotted on the world map. Similarly, genomes of ammonia-oxidizing archaea and bacteria (AOA and AOB), as well as comammox *Nitrospira* were retrieved from the NCBI, and different *amoA* and *amoB* genes were identified by comparing against PFAM models PF12942, PF02461, and PF04744 using HMMER [49] (v3.2.1). Near-complete *amoA* and *amoB* reads were mapped and filtered. Furthermore, the relative proportions of the different nitrifiers in metagenomes were determined by quantifying homologous reads for *amoA* or *amoB* genes.

### Comparative genome analysis

To elucidate the metabolic features of the newly recovered deep-sea MAGs (DS64 and DS176), comparative genome analysis was conducted across 296 *Nitrospirae* genomes. Genes of these genomes were clustered using OrthoFinder [50] (v2.4.0) with an e-values cutoff of 1e-5. For each protein family, a medoid sequence (defined as the sequence with the shortest summed genetic distances to all other family members) was predicted under the BLOSUM62 substitution matrix using the DistanceCalculator script in Phylo (https://biopython.org/wiki/Phylo). Medoid sequences were annotated by comparing against the COG [51], UniRef90 [52], and Pfam [53] databases using BLASTp [43] (v2.9.0) and HMMscan [49] (v3.2.1). The results for each gene cluster were further validated by cross-referencing all sequences against the COG and UniRef90 databases, with inconsistent annotation removed. Core clusters, including genes for nitrogen metabolism, stress adaptation, alternative energy metabolism, and the reductive tricarboxylic acid (rTCA) cycle, were identified based on the medoid sequence annotations.

## Results and discussion

### Genomic features and diversity of *Nitrospira* MAGs in deep-seamount sediments

**Figure 1:**
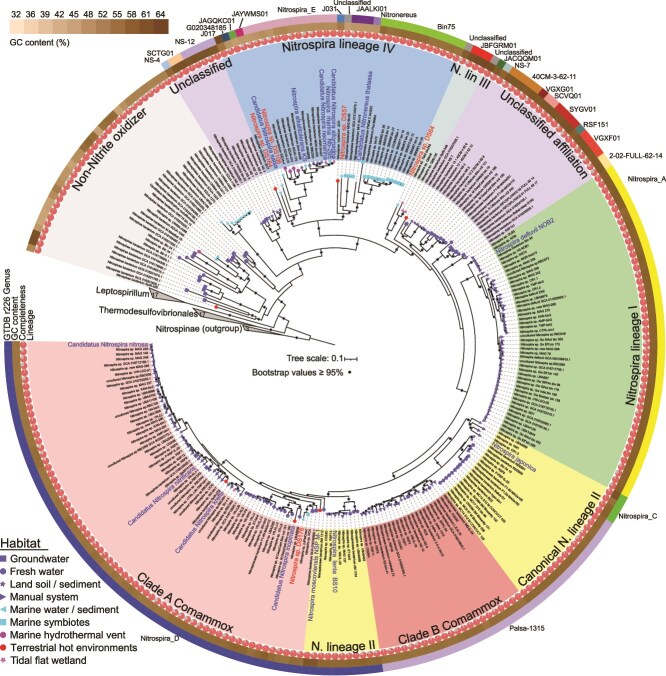
Phylogenomic affiliation of *Nitrospirae* genomes. The maximum-likelihood tree was constructed based on a concatenated set of 120 bacterial marker proteins from 304 genomes, including 279 *Nitrospirae* genomes from the NCBI and IMG databases, 14 cultured *Nitrospira* genomes (blue labels), five newly recovered *Nitrospira* metagenome-assembled genomes (red labels), and three *Nitrospinae* genomes (outgroup). Habitats for these *Nitrospira* species, including terrestrial ecosystems (groundwater, freshwater, soil, manual systems, and terrestrial hot environments [e.g. hot springs and engineered thermal systems]), marine environments (marine water, sediments, symbionts, and hydrothermal vents), and coastal wetlands, are indicated in the legend. *Nitrospira* lineages and sublineages are differentiated by sector colors. Bootstrap values ≥95% are indicated by black circles at nodes. Additional genomic features, including GTDB r226 genus taxonomy, GC content and genomic completeness, are provided in Supplementary Table S2.

Using a combination of binning and refinement tools, we recovered five *Nitrospira* MAGs (DS57, DS64, DS106, DS164, and DS176; with completeness >74% and contamination <4%) from 17 sediment samples collected from a deep-seamount region in the western Pacific Ocean (Supplementary Fig. S1; Supplementary Tables S1 and S2). Genome-wide ANI analysis revealed that these newly recovered *Nitrospira* MAGs represent distinct species, with all ANI values (73.5–86.5%) falling below the 95% species-level threshold [54] when compared both among themselves and with previously published *Nitrospira* genomes (Supplementary Table S3). Based on the latest release of GTDB (r226) [6], three of the recovered MAGs were classified into the following genera: *JAYWMS01* (DS106), *G020348185* (DS164), and *Nitrospira_D* (DS176), while the other two MAGs were assigned to the families *UBA8639* (DS57) and *Nitrospiraceae* (DS64) (Fig. 1; Supplementary Table S2). The genome size ranged from 2.2 to 5.1 Mbp, with GC content spanning 47.0% to 60.3% and the number of coding sequence (CDS) ranging from 2528 to 5686 (Supplementary Table S2).

To the best of our knowledge, only 16 genomes of lineage IV *Nitrospira* have been reported from open-ocean environments, including *N. marina*, Ca. *Nitrospira salsa* and Ca. *Nitronereus Thalassa* [10, 55, 56]. However, genomes of other *Nitrospira* lineages have not been reported from open-ocean environments. Our phylogenomic analysis, based on 120 concatenated marker proteins, identified three recovered MAGs (DPSM_bin57, DPSM_bin106, and DPSM_bin164) as members of lineage IV *Nitrospira*. In contrast, DS64 formed a distinct cluster with three MAGs derived from mangrove environments (TMG.bin248, QMm6, QMb135), and a full-length 16S rRNA gene sequence clustered with lineage III sequences from terrestrial ecosystems (Fig. 1; Supplementary Fig. S2) [40, 41]. The GC contents of these four lineage III *Nitrospira* MAGs (60.3–64.9%) were significantly higher than those of other *Nitrospira* lineage members (49.8–58.8%) [15]. Previously, members of lineage III *Nitrospira* were primarily identified in terrestrial ecosystems [41, 57], with only a single member previously reported in deep-sea sediments via 16S rRNA gene amplicon sequencing [58]. DS64 represents the first genomic evidence for lineage III *Nitrospira* in deep-sea ecosystems, providing critical insights into microbial biogeography. Moreover, the ANI values (74.6–74.8%) and RED value (RED <0.75) between DS64 and the mangrove-derived MAGs indicated that this evolutionarily distinct clade in deep-sea environments may possess unique adaptive strategies that enabling it to successfully cross marine–terrestrial boundaries (Supplementary Fig. S3; Supplementary Table S3).

In contrast, DS176 represents the first comammox *Nitrospira* MAG recovered from deep-sea ecosystems, with a genome size of 2.3 Mbp and GC content of 55.3% (Supplementary Table S2). The closest genomic relative in the database is *N.* sp. odPetCras1 recovered from a marine sponge metagenome (GCA_965247795.1, deposited in April 2025), with an ANI value of 95.7% (Supplementary Table S3) [54]. In the phylogenetic tree, these two marine-derived MAGs form a sister clade with *Nitrospira* sp. RCA (GCA_005239465.1), a clade A comammox *Nitrospira* MAG recovered from terrestrial subsurface groundwater in an aquifer adjacent to the Colorado River [59], with which they shared ANI values of 86.4–86.5% (Fig. 1; Supplementary Table S3). Furthermore, phylogenetic analysis of 16S rRNA gene showed a full-length sequence retrieved from the deep-sea sediment metagenomes clustered with those of cultured lineage II *Nitrospira* strains (Supplementary Fig. S2). These findings expand the known diversity of marine-derived lineage IV *Nitrospira*, and provide metagenomic evidence for the presence of lineage III and comammox *Nitrospira* in deep-sea habitats. Consequently, we further investigated the phylogenetic evolution, geographical distribution, and metabolic potential of the two newly identified deep-sea *Nitrospira* MAGs (DS64 and DS176).

### Phylogenetic affiliation of the novel *Nitrospira* MAGs based on functional gene analyses

Beyond species-level phylogenomic trees, functional gene-based phylogenetic analyses can further elucidate the evolutionary history of the newly discovered lineage III and comammox *Nitrospira* in deep-sea ecosystems. The *nxrB* gene, a reliable functional and phylogenetic marker for identifying uncultured NOB [40], was used to investigate the phylogenetic affiliations of the recovered MAGs. In the constructed *nxrB* phylogenetic tree, three full-length *nxrB* gene sequences (1290 bp) predicted from the contigs clustered with the *nxrB* gene sequence of the lineage III genome TMG248, with 91.3–92.3% nucleotide identity (Supplementary Fig. S4). Similarly, the *nxrB* sequence of DS176 clustered within the lineage II *Nitrospira*, showing 93.2–97.9% nucleotide identity with those of *N.* sp. RCA and *N.* sp. odPetCras1. These results demonstrate that the newly identified *Nitrospira* species possess the genetic potential for nitrite oxidation.

**Figure 2:**
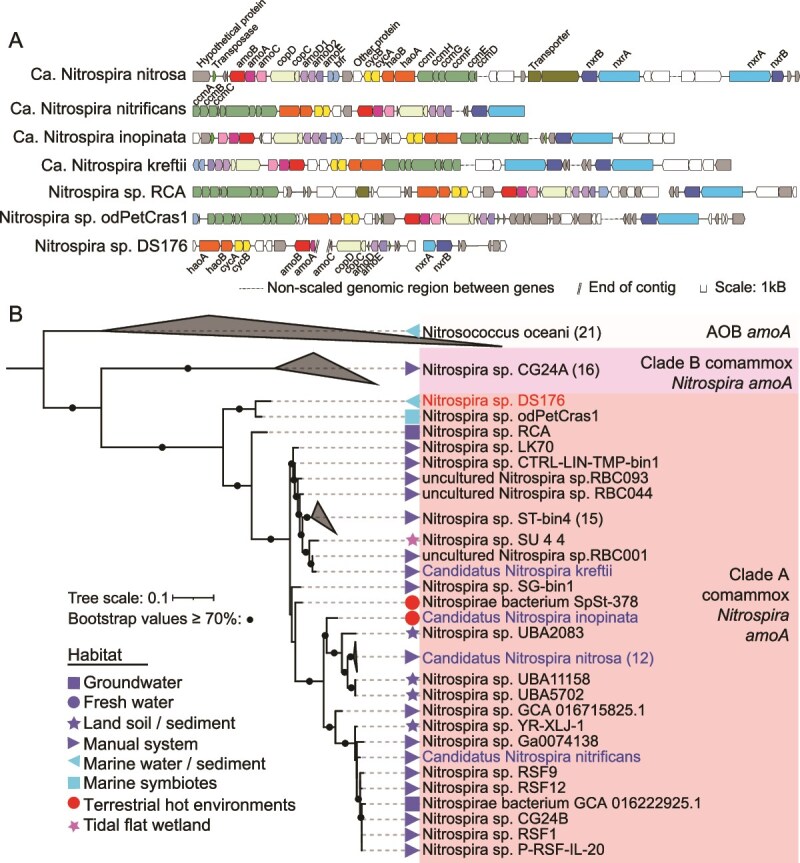
Gene loci and phylogeny of ammonia monooxygenase (AMO) in comammox *Nitrospira* genomes. (A) Schematic representation of AMO operons in seven comammox *Nitrospira* genomes. Arrows represent genes and their transcriptional direction. Predicted protein functions are shown with colors and tags. Abbreviations: *amoABCDE*, ammonia monooxygenase; *copCD*, copper transporter; *bfr*, bacterioferritin; *cycAB*, cytochrome c; *haoAB*, hydroxylamine dehydrogenase; *ccmABCDEFGHI*, cytochrome c biogenesis; *nxrAB*, nitrite oxidoreductase. All genes are drawn to scale. (B) Maximum-likelihood tree based on comammox and proteobacterial *amoA* amino acid sequences with LG + G4 substitution model and 1000 ultrafast bootstrap replicates. Proteobacteria *amoA* branch is set as the outgroup. Branches with bootstrap values >70% are marked with black circles. The number of sequences in each cluster is given in parentheses. The *amoA* sequences from DS176 were labeled in red, and those from cultured comammox species are in blue.

Unlike canonical *Nitrospira*, comammox *Nitrospira* obtain energy to support their chemoautotrophic growth by oxidizing ammonia to nitrate [3, 4]. The DS176 genome contains a complete gene set for AMO and HAO, which are necessary for the oxidation of ammonia to nitrite (Fig. 2A). Phylogenetic trees of *amoA* and *amoB* gene sequences showed that clade A comammox *Nitrospira* shared a common ancestor with clade B, and were closely related to those of β-proteobacterial AOB (β-AOB) (Fig. 2B; Supplementary Fig. S5) [3, 4]. This pattern likely results from horizontal gene transfer of the *amoAB* genes from β-AOB to comammox *Nitrospira*, followed by divergent evolution that enabled different clades to adapt to distinct ecological niches [60]. However, phylogenetic analyses of *haoA* and *haoB* gene sequences revealed that clade A comammox *Nitrispira* further divided into two sub-lineages (A1 and A2), with clade A2 sequences more closely related to clade B sequences (Supplementary Figs. S6 and S7). Notably, the *amoAB* and *haoAB* gene sequences of DS176 form a monophyletic cluster with those of marine sponge MAG *N.* sp. odPetCras1, with 95.6–98.8% amino acid identity. This finding highlights the evolutionary adaptation of comammox *Nitrospira* to marine habitats.

Synteny and gene locus analyses further provided critical insights for gauging the phylogenetic relationship among comammox species. Analogous to previously reported comammox genomes, the gene sets encoding AMO, HAO, and cytochrome c protein (CYC) in DS176 and *N.* sp. odPetCras1 are co-localized on contiguous contigs (Fig. 2A). The individual AMO operon and HAO-CYC operon consistently exhibit co-oriented arrangements with the order *amoCAB* and *haoAB*-*cycAB*, respectively. However, the relative orientation between the AMO and HAO-CYC operons varies. For example, in Ca. *Nitrospira nitrosa*, the operons are co-directionally arranged, whereas in other species, they exhibit a convergent orientation [59]. Furthermore, in DS176, the AMO operon is located downstream of the HAO operon, a configuration consistent with Ca. *N. nitrificans*, *N.* sp. RCA, *N.* sp. odPetCras1, but opposite to Ca. *N. inopinata* and Ca. *N. kreftii* (Fig. 2A). Intriguingly, transposase genes were identified upstream of the AMO operon in Ca. *Nitrosomonas nitrosa*, Ca. *N. inopinata* and *N.* sp. odPetCras1, suggesting high plasticity of the *amoCAB* gene cluster [3, 59]. A notable distinction of the marine-derived comammox species (*N.* sp. odPetCras1 and DS176) is the presence of only a single copy of the *amoD* and *amoE* genes, in contrast to other comammox members, which typically possess two copies of *amoD* and one copy of *amoE*. Here, the proximity of transposase genes to *amoD* in ocean-derived MAGs may provide genomic evidence supporting the duplication of the *amoD* gene in other comammox *Nitrospira* (Fig. 2A). Overall, synteny and gene loci analyses reveal distinct genomic and evolutionary features that differentiate deep-sea comammox *Nitrospira* from their terrestrial relatives, providing insights into their adaptation to marine ecosystems.

### Geographical distribution of the newly recovered MAGs in global ocean

**Figure 3:**
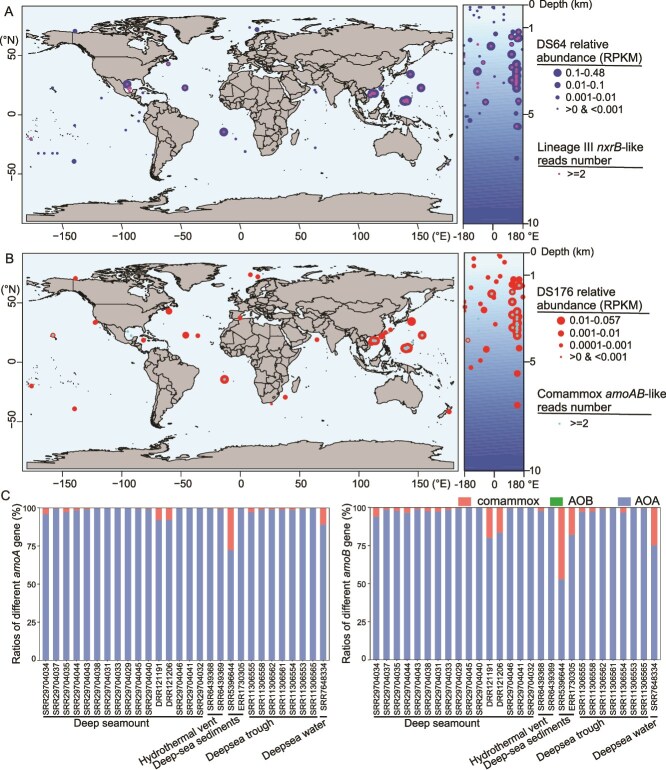
Global distribution of lineage III and comammox *Nitrospira* in marine environments. (A) Relative abundance and distribution of the lineage III *Nitrospira* genome (DS64) and the *nxrB* gene. The abundance of DS64 was quantified in metagenomic samples with ≥20 mapped reads, using reads per Kilobase per million mapped reads (RPKM), and is depicted as blue circles of proportional size to the RPKM values. The occurrence of lineage III *nxrB* sequences (≥2 mapped reads) is represented by pink circles (Supplementary Table S4). (B) Relative abundance and distribution of the comammox *Nitrospira* genome (DS176) and the *amoAB* genes. The RPKM abundance of DS176 (≥20 mapped reads) is illustrated with red circles, whereas the presence of *amoAB* sequences from comammox *Nitrospira* (≥2 mapped reads) is shown as cyan circles (Supplementary Table S5). (C) Relative proportions of *amoA* and *amoB* genes from various ammonia oxidizers across 48 marine samples, all of which contain comammox *amoA* or *amoB* gene homologues (Supplementary Table S5).

*Nitrospira* species are widely distributed in terrestrial ecosystems [12, 40], but studies on marine-derived *Nitrospira* remain limited. In this study, we identified two MAGs affiliated with lineage III (DS64) and comammox (DS176) *Nitrospira* from deep-sea environments for the first time. The global distribution of these MAGs was evaluated via read recruitment from 487 publicly available metagenomes, including 13 hydrothermal vent samples, 254 seawater samples, and 220 deep-sea sediment samples collected at depths ranging between 5 and 9161 m across global oceans (Supplementary Tables S4 and S5). Among these 487 metagenomes, 133 contained homologous sequences (read counts ≥20) of DS64, with a pan-oceanic distribution across the Pacific and Atlantic Oceans (Fig. 3A; Supplementary Table S4). Although lineage III *Nitrospira* homologues were detected in 44.5% of deep-sea sediment metagenomes and 28.6% of deep-sea water metagenomes, they exhibited relatively low abundance as indicated by low RPKM value (<0.1) in most samples. This scarcity likely explains why lineage III *Nitrospira* genomes have not been binned in previous studies. However, RPKM values for the MAG DS64 were significantly higher in deep-sea sediments, hydrothermal vent and deep-sea waters (8.6E-2, 5.4E-2 and 5.1E-4, respectively; given as mean values, as mentioned below) than in epipelagic and mesopelagic zone waters (5.0E-5 and 7.4E-5, respectively; *P* < .01; Fig. 3A; Supplementary Table S4), suggesting a specific niche preference for deep-sea environments.

**Figure 4:**
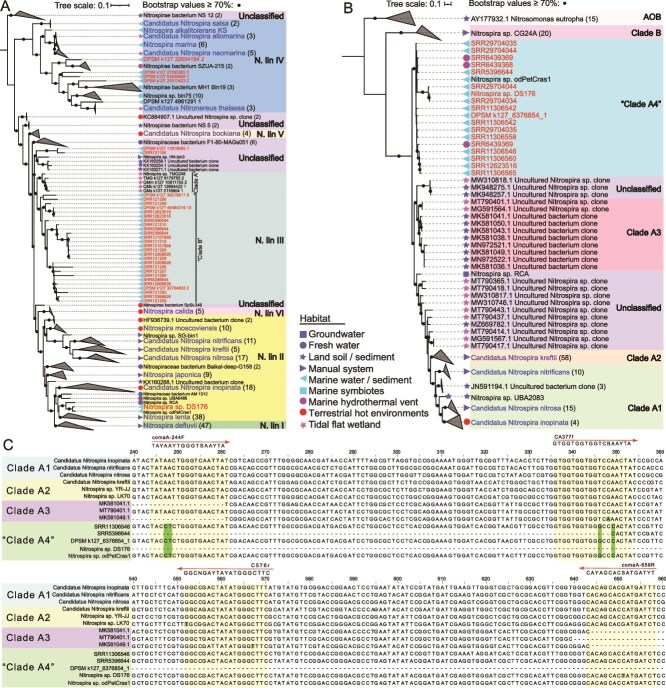
Phylogenetic trees based on *nxrB* and *amoA* homologues and alignment of comammox *amoA* nucleotide sequences. (A) The maximum-likelihood (ML) phylogenetic tree was constructed based on 255 *Nitrospira nxrB* gene sequences (>1000 bp) with the GTR + F + R5 model and 1000 ultrafast bootstrap replicates. The *nxrB* sequences obtained from marine metagenomes are labeled in red. Branches with bootstrap values >70% are indicated with black circles. (B) The ML tree was constructed based on 162 amoA gene sequences from comammox *Nitrospira* and ammonia-oxidizing bacteria with the TIM3 + F + R4 model and 1000 ultrafast bootstrap replicates. Branches with bootstrap values >70% are indicated with black circles. (C) Nucleotide sequences alignment of *amoA* gene from representative members of clades A1–A4 comammox *Nitrospir*a. The numbers above the sequences represent aligned nucleotide positions. Two commonly used primer pairs, amoA-244F/amoA-659R and CA377F/C576R, are annotated above their respective binding regions. Non-conserved sites within these primer-binding regions are marked with green shading.

The global distribution and relative abundance of lineage III *nxrB* genes were further evaluated based on the three full-length *nxrB* gene sequences identified from metagenomes. Comparative read recruitment revealed that lineage III *nxrB* gene was exclusively detected in deep-sea sediment and hydrothermal vent metagenomes, with CPM values of 1.21 and 0.98, respectively. A significant correlation between the abundance of an *nxrB* sequence (k127_32704833) and DS64 (*P* < 2.2E–16, *R* = 0.81; Supplementary Fig. S8) suggests that DS64 is the most likely host for this sequence. Phylogenetic analysis of *nxrB* gene sequences, assembled from the aforementioned metagenomes, showed that most of marine-derived sequences clustered with the three *nxrB* sequences (k127_32704833, k127_46484319, and k127_35519917), and were evolutionarily distinct from those detected in coastal wetland metagenomes. The lineage III *nxrB* gene sequences from coastal wetland exhibited a minimum nucleotide dissimilarity of 7% relative to those from oceans based on near-full-length sequences, and we therefore designate these two groups as “Clade A” and “Clade B,” respectively (Fig. 4A). Previous studies have reported lineage III species in terrestrial cave systems [41, 57] and deep-sea sediments [58] based on 16S rRNA gene amplicons. Our findings significantly expand the known diversity and functional potential of lineage III *Nitrospira* species.

Although comammox *Nitrospira* are widespread in engineered systems, soils, freshwater environments, and coastal ecosystems [12, 61], their marker genes or MAGs had not been detected in deep-sea environments. Of the 487 metagenomes, 100 contained homologous sequences (read counts ≥20) of DS176, showing a wide distribution in the Pacific and Atlantic Oceans (Fig. 3B; Supplementary Table S5). However, the abundance of DS176 homologues was approximately one order of magnitude lower than that of DS64, with most samples exhibiting RPKM values below 0.01. Similarly to DS64, DS176 was predominantly detected in deep-sea environments, with significantly higher abundance in deep-sea sediments, hydrothermal vent and deep-sea waters (1.1E-2, 6.8E-3 and 2.3E-4, respectively) compared to epipelagic and mesopelagic zone waters (1.2E-4 and 1.3E-4, respectively; *P* < .01; Supplementary Table S5).

The environmental distribution of DS176 was further evaluated by mapping *amoA* and *amoB* gene sequences from the NCBI database to the metagenomic dataset. Comammox *amoA* and *amoB* genes were detected in 21 and 22 metagenomes (read counts ≥2), respectively, with average CPM values of 7.6E-2 and 1.0E-1 (Fig. 3B). The abundance of comammox *amoA* and *amoB* genes was significantly lower than that from AOA, but higher than that from AOB (Fig. 3C; Supplementary Table S5), suggesting that comammox may contribute more substantially to ammonia oxidation than AOB in certain habitats. However, no homologs of comammox *amoA* and *amoB* genes were detected in 150 epipelagic and 40 mesopelagic water metagenomes. This vertical distribution may be attributed to both positive adaptation and negative inhibition. On one hand, canonical comammox *Nitrospira* have a high affinity for ammonia [62], enabling them to thrive in oligotrophic oceans. DS176 and *N.* sp. odPetCras1 formed a monophyletic clade with two strains from groundwater, indicating their adaptations to oxygen availability and hydrostatic pressure [9]. On the other hand, these comammox *Nitrospira* in the upper oceanic zones may experience photoinhibition of ammonia oxidation [63] and face competition from AOA and other planktonic microorganisms for ammonia and nitrite substrates [64].

Phylogenetic analysis of *amoA* sequences assembled from metagenomes showed that the *amoA* sequence of DS176 clustered with other marine-derived comammox *amoA* sequences (Fig. 4B). These sequences exhibited a minimum nucleotide dissimilarity of 15% compared to sequences of clade A1–A3 found in terrestrial and coastal ecosystems [17, 18, 65, 66], and we designate this marine-derived group as “clade A4.” Sequence alignment revealed that the conserved sites of the two primer pairs used for amplifying previously characterized comammox *amoA* genes were not conserved in clade A4 (Fig. 4C). Specifically, nucleotide substitutions in primer-binding regions (i.e. A247 → C247 and A248 → T248 for the comaA-244F primer [67]; T346 → G346 and A349 → C349 for the CA377f primer [68]) indicate mismatches that likely impede detection of clade A4 comammox *Nitrospira* in deep-sea sediments. Three *amoA* clone sequences in an adjacent branch were retrieved from estuarine and tidal flat wetlands (Fig. 4B), suggesting that coastal and deep-sea clades may have evolved from a saline-adapted common ancestor.

### Important metabolisms of two novel MAGs and environmental adaptation revealed by comparative genomic analysis

**Figure 5:**
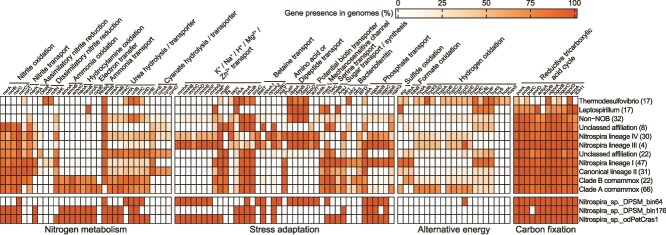
Metabolic features of genomes within different *Nitrospira* lineages and sublineages. The MAGs recovered in this study are shown in the lower panel. The percentage of each gene was quantified in the genomes of each group, and results are indicated in the heatmap (Supplementary Table S6). The number of genomes in each group is indicated in brackets (Supplementary Table S2). Abbreviations: *nxrABC*, nitrite oxidoreductase; *nirC*, nitrite transporter; *nark*/*nasA*, nitrite/nitrate transporter; *nirA*, assimilatory nitrite reductase; ONR, octaheme cytochrome c nitrite reductase; *nrfAH*, cytochrome c nitrite reductase; *nirK*, dissimilatory nitrite reductase; *amoABC*, ammonia monooxygenase; *haoAB*, hydroxylamine dehydrogenase; *cycAB*, cytochrome c; *Rh50*, Rh-type ammonium transporter; *amtB*, ammonium transporter; *ureABC*, urease; *urtABCD*E, ABC-type urea transport system; *cynS*, cyanate lyase; *cynABD*, cyanate ABC transporter; *mnhABCDEFG*, multi-subunit Na^+^/H^+^ antiporter; *NhaP*, Na^+^/H^+^ antiporter; *mgtE*, magnesium transporter; *trkAH*, K^+^ transport system regulatory component; *znuABC*, ABC-type zinc transport system; *BetT*, choline/glycine/proline betaine transport protein; *opuABC*, glycine/betaine ABC-type transporter; *AlsT*, Na+/alanine symporter; *PutP*, Na^+^/proline symporter; *dppABCD*, dipeptide transport; *ecfTA*, energy coupling factor transporter; *mscL*, mechanosensitive channel; *YbeC*, serine transporter; *melB*, Na^+^/melibiose symport; *AraJ*, arabinose efflux permease; *bfr*, bacterioferritin; *BFD*, bacterioferritin-associated ferredoxin; *pitA*, phosphate transport; *ugpABCEQ*, ABC-type glycerol-3-phosphate transport system; *sqr*, sulfide:quinone oxidoreductase; *fdhAB*, formate dehydrogenase; *hypABCDEF* and *hyaD*, hydrogenase accessory protein; *hyfCEFGI*, putative group 4 hydrogenase; *hybD* and *hydABDG*, group 3 sulfur-reducing hydrogenase; *hupSL*, group 2a [Ni-Fe] hydrogenase; *porABCD*, pyruvate:ferredoxin oxidoreductase; *aclyAB*, ATP citrate lyase; *ogorABCD*, 2-oxoglutarate:ferredoxin oxidoredutase; *acnA*, aconitate hydratase; *IDH1*, isocitrate dehydrogenase.

*Nitrospira* species are known for their high metabolic diversity [56, 69, 70]. Based on phylogenomic analysis, they are divided into eight distinct groups (Fig. 1), including the canonical lineages I-IV, the comammox clades A and B, and two unclassified clades. To investigate the unique metabolic properties and environmental adaptations of the two newly recovered deep-sea *Nitrospira* genomes (DS64 and DS176), we conducted comparative genomic analysis across publicly available genomes of the *Nitrospirae* phylum (Fig. 5; Supplementary Table S6). The membrane-anchored molybdopterin-binding NXR enzyme is a key signature for nitrite-oxidizing *Nitrospira* [5]. DS64, a lineage III *Nitrospira*, contains only one gene (*nxrC*) encoding the NXR γ-subunit, which, however, is likely attributable to incomplete genome recovery. DS176 and the marine sponge MAG *N.* sp. odPetCras1 contain all NXR subunits, as well as the full gene set of AMO, which is essential for ammonia oxidation (Fig. 5). Additionally, DS176 encodes the HAO-CYC operon (*haoAB*-*cycAB*) [3, 71], which is absent in canonical *Nitrospira* (Fig. 5).

As oligotrophic environments, deep-sea waters and sediments are typically characterized ammonia- and nitrite-limited conditions, due to limited organic input and microbial activity [72]. Although genes encoding nitrite transporters (*NirC* and *NarK*) were not detected in either DS64 or DS176, the presence of periplasmic-oriented NXR and its high nitrite affinity (*K_m_* = ~ 9–27 μM) suggests that these *Nitrospira* can operate under nitrite-limited conditions [5, 19]. Like other *Nitrospira* genomes, most comammox *Nitrospira*, including *N.* sp. odPetCras1, encode a copper-dependent nitrite reductase (*nirK*) for detoxifying excess nitrite or for operating in reverse to further oxidize NO formed by HAO to nitrite [73–75]. Intriguingly, both DS176 and *N.* sp. odPetCras1 appear to utilize cytoplasmic ferredoxin-dependent nitrite reductase (*nirA*) for nitrite assimilation, which is absent in previously reported comammox *Nitrospira* (Fig. 5) [59].

Regarding ammonium uptake, DS64, like other lineage III and IV *Nitrospira*, encodes *amtB*-type ammonium transporters that enable direct ammonium uptake for assimilation [56]. Previous studies have shown that clade A comammox *Nitrospira* encode *Rh50*-type ammonium transporters, in contrast to clade B members, which rely on *amtB*-type ammonium transporters [60, 76]. However, DS176, *N.* sp. odPetCras1, and *N.* sp. RCA, along with most members of clade B comammox *Nitrospira*, lack the *Rh50* gene but harbor three copies of *amtB* homologues (45–51% amino acid identity to *Nitrospira moscoviensis*; Supplementary Fig. S9). This divergence may result from niche separation among clade A comammox *Nitrospira*, as *amtB*-type transporters exhibit higher ammonium affinity but lower uptake capacity than *Rh50*-type transporters [60]. This finding aligns with observations that clade B comammox *Nitrospira* and *amtB*-encoding clade A members are predominantly found in oligotrophic environments—such as nitrogen-deficient forest soils, open oceans, and groundwater [59, 77]—characterized by environmental scarcity and cultivation challenges [12]. The *Rh50* gene in other clade A comammox *Nitrospira* may have originated through horizontal gene transfer from β-AOB, and its acquisition may be associated with *amtB* gene loss under eutrophic conditions [78, 79]. In oligotrophic marine environments, urea may play an important role in supporting ammonium and nitrite oxidation [80]. DS64, DS176, and *N.* sp. odPetCras1 encode genes for urease (*ureABC*) and urea transporters (*urtABCDE*), enabling the utilization of urea as an alternative energy source, thereby supporting ecosystem functions by facilitating the cycling of nitrogen and organic matter in the deep sea [81]. However, genes encoding cyanase (*cynS*), which catalyzes the conversion of cyanate to ammonia, are absent in DS176 and *N.* sp. odPetCras1, consistent with other comammox *Nitrospira* genomes (Fig. 5).

Microorganisms living in marine environments must be capable of coping with the high salinity of seawater. Genes encoding the multi-subunit Na^+^/H^+^ antiporter (*mnh*), which are used to maintain cytoplasmic pH and provide salt tolerance, are widespread in Lineage III (including DS64) and IV *Nitrospira*, but absent in lineage I and II (Fig. 5). However, *N.* sp. odPetCras1, a species affiliated with clade A comammox *Nitrospira*, harbors *mnh* genes, which may have originated via horizontal gene transfer from lineage III or IV *Nitrospira* (Supplementary Figs. S10–S12). The presence of these genes likely plays a crucial role in enabling the newly discovered lineage III and comammox *Nitrospira* microbes to tolerate the highly saline seawater. In addition, *N.* sp. odPetCras1 encodes a single-gene type Na^+^/H^+^ antiporter (*NhaP*), further enhancing its capacity to thrive in hypersaline marine environments. Notably, the absence of these genes in DS176 is most likely due to the incomplete recovery of the genome. Betaine is an important microbial osmolyte, and DS64, DS176, and *N.* sp. odPetCras1 encode genes for choline/glycine/proline betaine transporters (*BetT*, *opuABC*) [10, 82], which are uncommon in members of lineage I and II *Nitrospira* (Fig. 5; Supplementary Figs. S13–S14). In addition, like other members of lineage III *Nitrospira*, DS64 encodes additional organic solute transport systems, including a sodium:alanine symporter (*AlsT*), a serine transporter (*YbeC*), and an ABC-type dipeptide transporter (*DdpA* and *DppBCD*), which could be useful for regulating cellular osmotic pressure in seawater. Jiang et al. [24] recovered an MAG Nitrospira.bin98 (clustered with Ca. *N. kreftii*, a clade A comammox *Nitrospira* species), containing genes encoding a Na^+^-translocating N-ATPase (*atp*) and a sodium-pumping complex I (*nqr*), thereby exhibiting a certain level salt tolerance and enabling its survival in brackish ecosystems such as salt marshes and mangroves [22, 23]. However, the absence of *mnh* genes in Ca. *N. kreftii* may explain its inability to thrive in open-ocean environments.

For utilization of phosphorus, DS64, DS176, and *N.* sp. odPetCras1 harbor genes encoding a phosphate transporter (*pitA*) and an ABC-type glycerol-3-phosphate transport system (*ugpABCE*). The *ugpABCE* operon is a prevalent system in the open ocean that enables microbes to efficiently take up monoesters and diesters of glycerol phosphate in phosphorus-deficient environments [83], but it is rarely found in known comammox *Nitrospira* genomes. Phylogenetic analysis revealed that the *ugpA* gene of DS176 does not cluster within a monophyletic group with homologous genes from other comammox clade A genomes, suggesting it may have been acquired through horizontal gene transfer from other marine microorganisms (Supplementary Fig. S15). In addition, DS64 encodes genes for coupling factor transporters (*EcfA* and *EcfT*), which facilitate the extracellular uptake of biotin [84]. DS64 and *N.* sp. odPetCras1 encode a gene (*CbiA*) for exopolysaccharide biosynthesis (Supplementary Table S6), indicating their potential for a biofilm-associated lifestyle [85]. However, both DS64 and DS176 contain few genes involved in the utilization of alternative electron donors, such as sulfide, formate, and hydrogen, suggesting a heavy reliance on nitrification reactions for energy acquisition. Core genes encoding enzymes for the rTCA cycle, including ATP-citrate lyase, 2-oxoglutarate:ferredoxin oxidoreductase and pyruvate ferredoxin oxidoreductase, were identified in DS64 and DS176, indicating that these species employ the rTCA cycle for CO_2_ fixation in the deep-sea environments.

Collectively, phylogenomic, phylogenetic, and comparative genomic analyses indicate that marine-derived comammox *Nitrospira* (including DS176 and *N.* sp. odPetCras1) diverged early within the branch of clade A comammox *Nitrospira*. These species may have acquired *mnh* and *ugp* genes from other microorganisms, enabling them to combat the saline and oligotrophic marine environments. However, An alternative scenario is that clade A comammox *Nitrospira* may have originated in open-ocean environments, with subsequent evolutionary adaptations leading to terrestrial species such as *N.* sp. RCA (groundwater) and Ca. *N. kreftii* (estuarine water). During this transition, genes such as *betT*, *opuABC*, and *amtB* may have been gradually lost, whereas the *Rh50* gene was acquired via horizontal gene transfer, facilitating adaptation to eutrophic terrestrial water environments.

## Conclusion

In summary, this study recovered five novel *Nitrospira* MAGs from deep-sea sediments and provides metagenomic evidence for the presence of lineage III and comammox *Nitrospira* in deep-sea environments. Phylogenomic analysis revealed that both marine lineage III and comammox *Nitrospira* are distinct from their terrestrial counterparts. Notably, the early divergence of marine comammox clade A indicates a long evolutionary trajectory shaped by the unique physicochemical conditions of marine environments. Furthermore, our analysis suggested that lineage III and comammox *Nitrospira* are potentially distributed throughout the deep ocean, with significantly higher abundances in deep-sea sediments and waters compared to the epipelagic and mesopelagic zones. This niche specialization suggests that these microorganisms play a previously underappreciated role in nitrogen cycling under the extreme substrate-limited, oxygen-constrained, and high-pressure conditions characteristic of the deep sea. Metabolic features identified in these two novel MAGs, such as adaptations to oligotrophic and saline seawater environments, as well as capabilities supporting chemoautotrophic growth, further corroborate their ecological fitness and potential for pan-oceanic distribution in deep-sea habitats. Collectively, this study not only expands the current understanding of *Nitrospira* diversity in the ocean, but also provides new perspectives on the evolutionary history of various *Nitrospira* lineages. Although the evidence from MAGs presented here confirms the presence of lineage III and comammox *Nitrospira* in the deep ocean, follow-up studies will be needed to further validate these findings through enrichment cultivation or single-cell genomic approaches. Such efforts would not only validate the metabolic capabilities inferred from genomic data but also enable direct physiological characterization and *in situ* activity measurements, thereby clarifying their ecological roles in the deep-sea nitrogen cycle.

## Supplementary Material

Supplementary_Materials_ycag003

## Data Availability

Genome bins and sequences used for phylogenetic analyses are available on Figshare (https://figshare.com/s/ad75994a04913eb1f5c9) or the NCBI database under BioProject ID PRJNA1300331 (https://www.ncbi.nlm.nih.gov/bioproject/PRJNA1300331), with accession numbers ranging from SAMN50335977 to SAMN50335981. Raw sequencing reads can be accessed via the NCBI Sequence Read Archive under BioProject ID PRJNA1131620 (https://www.ncbi.nlm.nih.gov/bioproject/PRJNA1131620), with accession numbers ranging from SRR29704029 to SRR29704047. All data required to evaluate the conclusions are provided in this paper and/or the Supplementary Materials.
