# Supplementary material for: Unique phylogenies and metabolic adaptations of novel lineage III and comammox Nitrospira species from deep-sea sediments
Source: ISME Commun. 2026 Jan 9;6(1):ycag003. doi: 10.1093/ismeco/ycag003 (PMC12903955; doi:10.1093/ismeco/ycag003)
Supplement: Supplementary_Materials_ycag003 [file supplementary_materials_ycag003.zip › Supplementary Information.pdf]

Supplementary information for

Unique phylogenies and metabolic adaptations of novel lineage III and comammox *Nitrospira* species from deep-sea sediments

Guohao Chen[1,‡], Hongmei Jing[2,‡], Bolin Liu[1,*], Jiawei Zhang[1], Yafei Ou[1], Wenxiao Liu[1], Xinru Tian[1], Ran Wang[1], Jinlin Yan[1], Tieqiang Mao[1], Sai Yang[1], Yanling Zheng[3], Lijun Hou[1,*], Hongpo Dong[1]

[1]State Key Laboratory of Estuarine and Coastal Research, Yangtze Delta Estuarine Wetland Ecosystem Observation and Research Station, East China Normal University, Shanghai, China

[2]CAS Key Laboratory for Experimental Study Under Deep-Sea Extreme Conditions, Institute of Deep-Sea Science and Engineering, Chinese Academy of Sciences, Sanya, China

[3]Key Laboratory of Geographic Information Science of the Ministry of Education, School of Geographic Sciences, East China Normal University, Shanghai, China

*Corresponding authors: Bolin Liu, State Key Laboratory of Estuarine and Coastal Research, East China Normal University, 500 Dongchuan Road, Shanghai 200241, China. Email: blliu@sklec.ecnu.edu.cn; Lijun Hou, State Key Laboratory of Estuarine and Coastal Research, East China Normal University, 500 Dongchuan Road, Shanghai 200241, China. Email: ljhou@sklec.ecnu.edu.cn

‡These authors contributed equally to this work.

Supplementary Methods

NxrB gene sequences phylogeny

A total of 10 *Nitrospira nxrB* gene sequences were identified from deep-seamount metagenome-assembled genomes (MAGs) or metagenomes using Prodigal [1] (v2.6.3) and BLASTn [2] (v2.9.0). Additionally, 151 *nxrB* sequences were obtained from other *Nitrospira*-like genomes or metagenomes, and 68 sequences were retrieved from the NCBI database (Supplementary Table S2) [3]. The combined *nxrB* gene sequences were aligned using MAFFT [4] (v7.402) and trimmed with BMGE [5] (v1.12). A maximum likelihood (ML) phylogenetic tree was constructed using IQ-TREE [6] (v1.6.12) with the GTR+F+R5 model and 1 000 ultrafast bootstrap replicates.

AmoA and *amoB* gene sequences phylogeny

The *amoA* and *amoB* gene sequences from DS176 were identified by comparing against the NCBI nr database using BLASTp [2] (v2.9.0). Additional *Nitrospira amoA* and *amoB* sequences were either retrieved from the NCBI database or extracted from *Nitrospira* genomes using HMMER [7] (v3.2.1) with the PFAM models PF02461 and PF04744. Proteobacterial *amoA* and *amoB* sequences, obtained from the NCBI database, were used as the outgroup. The combined sets of *amoA* or *amoB* amino acid sequences were aligned using MAFFT [4] (v7.402) and trimmed with BMGE [5] (v1.12). ML phylogenetic trees for *amoA* and *amoB* genes were constructed using IQ-TREE [6] (v1.6.12) with the best-fit models (LG+G4 and LG+R4, respectively) and 1 000 ultrafast bootstrap replicates.

HaoA and *haoB* gene sequence phylogeny

The *haoA* and *haoB* gene sequences from MAG DS176 were identified by comparing against the NCBI nr database using BLASTp [2] (v2.9.0). Other comammox *haoA* and *haoB* sequences were identified from retrieved comammox genomes (Supplementary Table S2). Proteobacterial *haoA* and *haoB* sequences were used as the outgroup. The *haoA* and *haoB* amino acid sequences were aligned using MAFFT [4] (v7.402) and trimmed with BMGE [5] (v1.12). ML phylogenetic trees for *haoA* and

haoB genes were constructed using IQ-TREE [6] (v1.6.12) with the best-fit models (LG+R4 and JTT+I+G4, respectively) and 1 000 ultrafast bootstrap replicates.

NxrB and *amoA* homologues phylogeny

To address the unclear phylogeny of recruited read homologues for lineage III *nxrB* and comammox *amoA* genes, we assembled these short reads using SPAdes [8] (v3.15.2) and retained only the successfully assembled contigs for subsequent phylogenetic analysis. Reference *nxrB* and *amoA* gene sequences from previous studies [3, 9] were used for the respective phylogenetic analyses of these two genes. The *nxrB* and *amoA* gene sequences were aligned using MAFFT [4] (v7.402) and trimmed with BMGE [5] (v1.12). ML phylogenetic trees for *nxrB* and *amoA* genes were constructed using IQ-TREE [6] (v1.6.12) with the best-fit models (GTR+F+R5 and TIM3+F+R4, respectively) and 1 000 ultrafast bootstrap replicates.

16S rRNA gene sequence phylogeny

The 16S rRNA gene sequences were predicted from the assembled contigs using Barrnap (https://github.com/tseemann/barrnap; v0.9), and subsequently compared against a database containing cultured *Nitrospira* 16S rRNA gene sequences using BLASTn [2] (v2.9.0). Based on a 90% nucleotide identity threshold, three near-full-length (>1 000 bp) *Nitrospira*-like 16S rRNA gene sequences were recovered. To further elucidate their phylogeny, we retrieved 184 *Nitrospira* 16S rRNA gene sequences from the NCBI database as described by Pester et al. [3], and predicted an additional 83 sequences from the *Nitrospirae* genomes (Supplementary Table S2). The combined sets of 269 *Nitrospira* 16S rRNA gene sequences was aligned using MAFFT [4] (v7.402), and trimmed with BMGE [5] (v1.12). An ML phylogenetic tree was constructed using IQ-TREE [6] (v1.6.12) with the TNe+R5 model and 1 000 ultrafast bootstrap replicates.

MnhABC, *opuABC*, and *ugpA* gene sequences phylogeny

The genes encoding the multi-subunit Na^+/H^+ antiporter (*mnh*), the glycine/betaine ABC-type transporter (*opu*), and ABC-type glycerol-3-phosphate transport system (ugp) are key marker genes for *Nitrospira* species to be capable of coping with the high saline

and phosphorus-limited seawater, and phylogenetic analyses were performed on the genes encoding various subunits of these transporters. Amino acid sequences for *mnhA*, *mnhB*, *mnhC*, *opuA*, *opuBC*, and *ugpA* were identified from retrieved *Nitrospirae* genomes (Supplementary Table S2) based on clustered protein families (Supplementary Table S6). Additionally, 11 *opuA* amino acid sequences were retrieved from the NCBI database. Each set of sequences corresponding to different subunits was aligned using MAFFT [4] (v7.402) and subsequently trimmed with BMGE [5] (v1.12). ML phylogenetic trees were constructed using IQ-TREE [6] (v1.6.12) with the best-fit models and 1 000 ultrafast bootstrap replicates.

Supplementary Figures

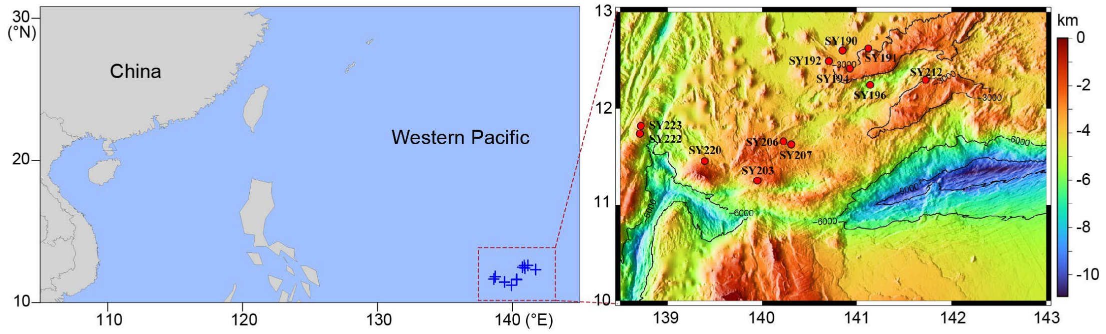

Fig. S1. Station and topographic map of deep-seamount regions in the western Pacific Ocean. The "+" symbols and red circles denote the sampling sites.

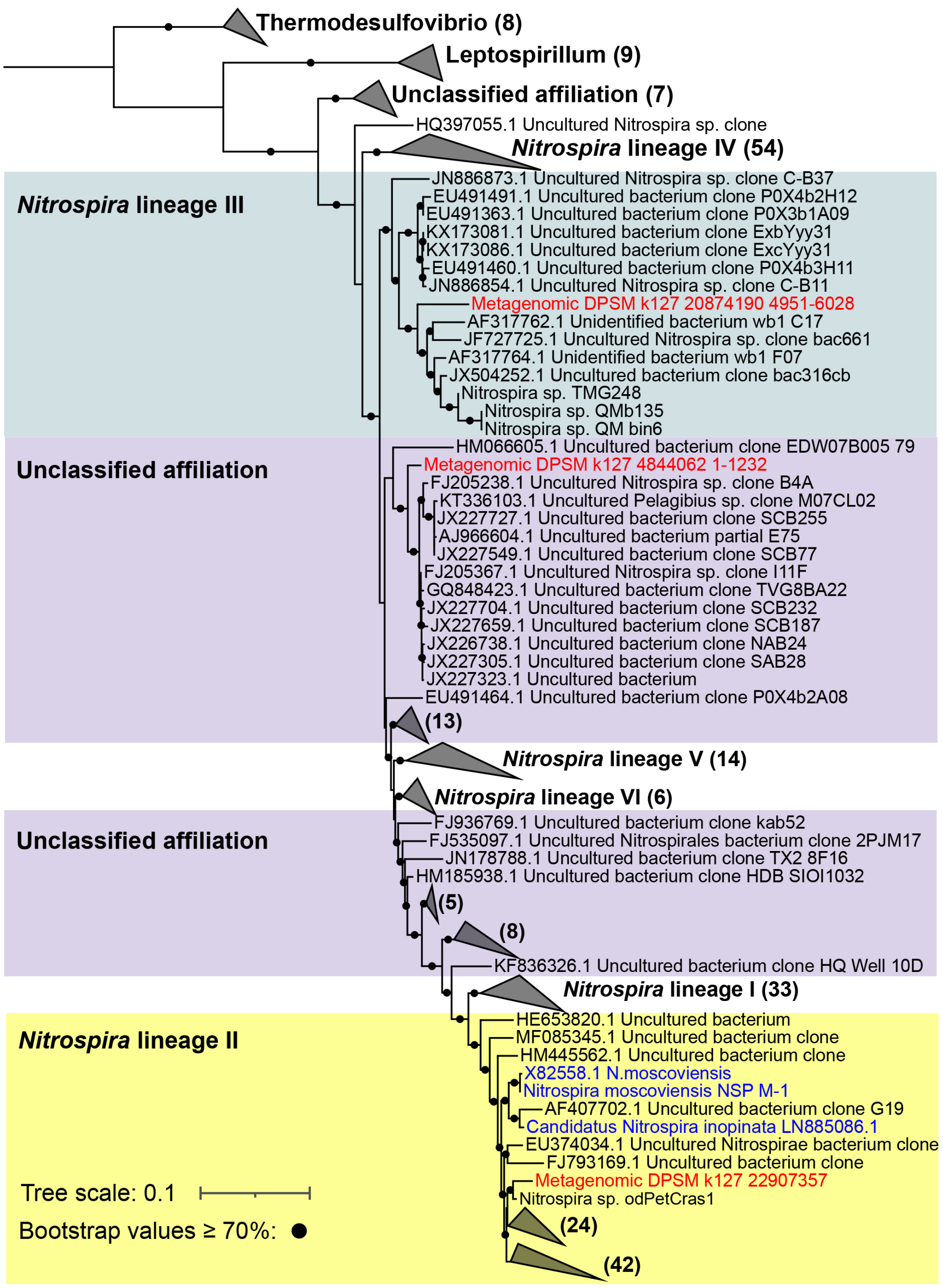

Fig. S2. Phylogenetic tree based on 16S rRNA gene sequences of the *Nitrospira*e phylum. The maximum likelihood tree was constructed using 270 16S rRNA gene sequences, including 253 sequences retrieved from *Nitrospira*-like species and 17 sequences from the broader *Nitrospira*e phylum, with the TNe+R5 model and 1 000 ultrafast bootstrap replicates. The 16S rRNA gene sequences of *Thermodesulfovibrio* were used as the outgroup. Sequences derived from deep-sea metagenomes in this study were labeled in red, and those from cultured *Nitrospira* strains were labeled in blue. Branches with bootstrap values ≥70% are indicated with black circles.

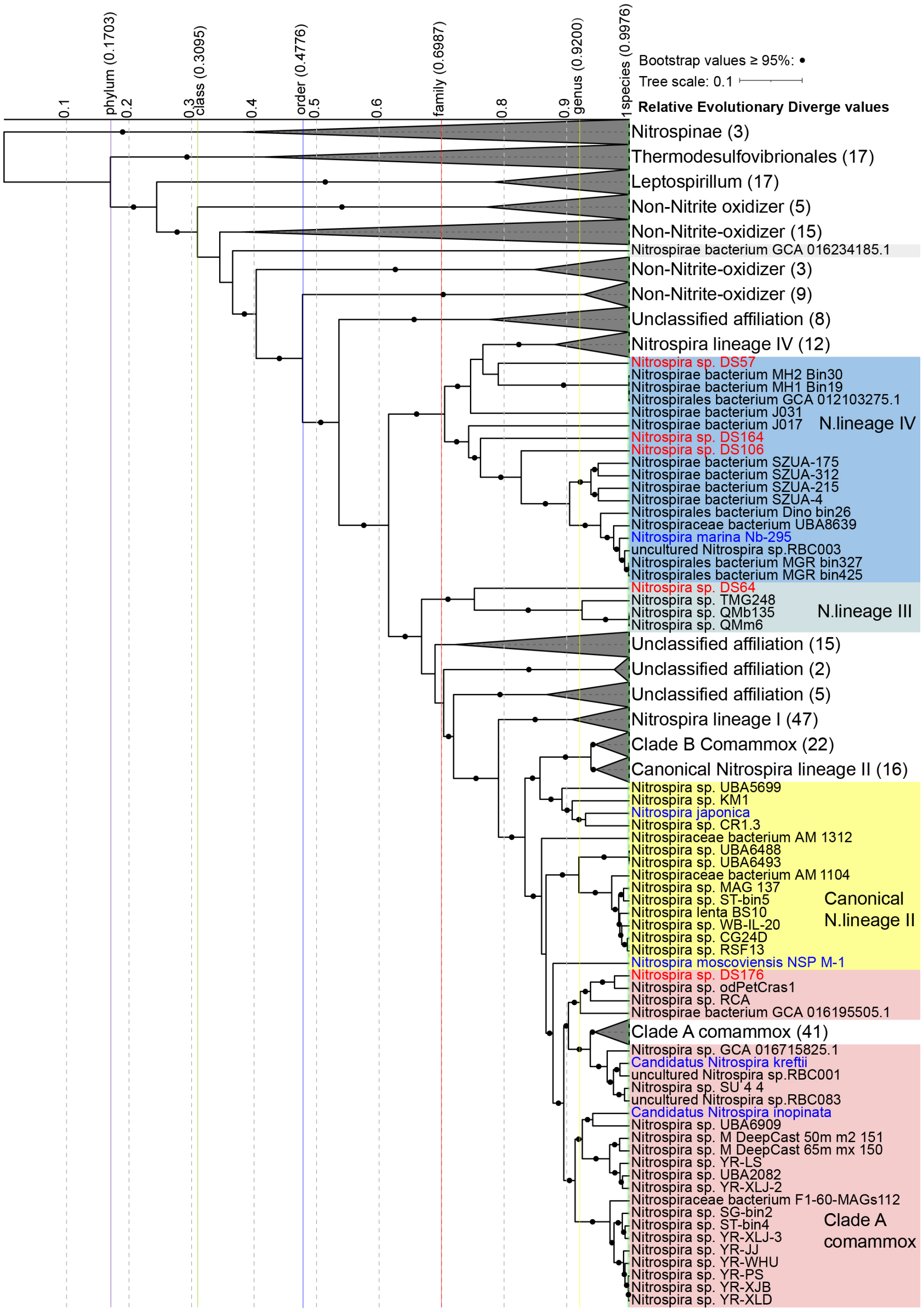

Fig. S3. Relative evolutionary divergence (RED) of species in the *Nitrospirae* phylum. The RED values for 296 *Nitrospirae* genomes are represented by branch lengths, based on the GTDB taxonomy. Taxonomic labels at different levels are shown in varying colors along the internal tree scale. Branches with bootstrap values ≥ 95% are indicated with black circles. The metagenome-assembled genomes (MAGs) recovered from deep-sea metagenomes in this study were labeled in red, and species of cultured *Nitrospira* were marked in blue.

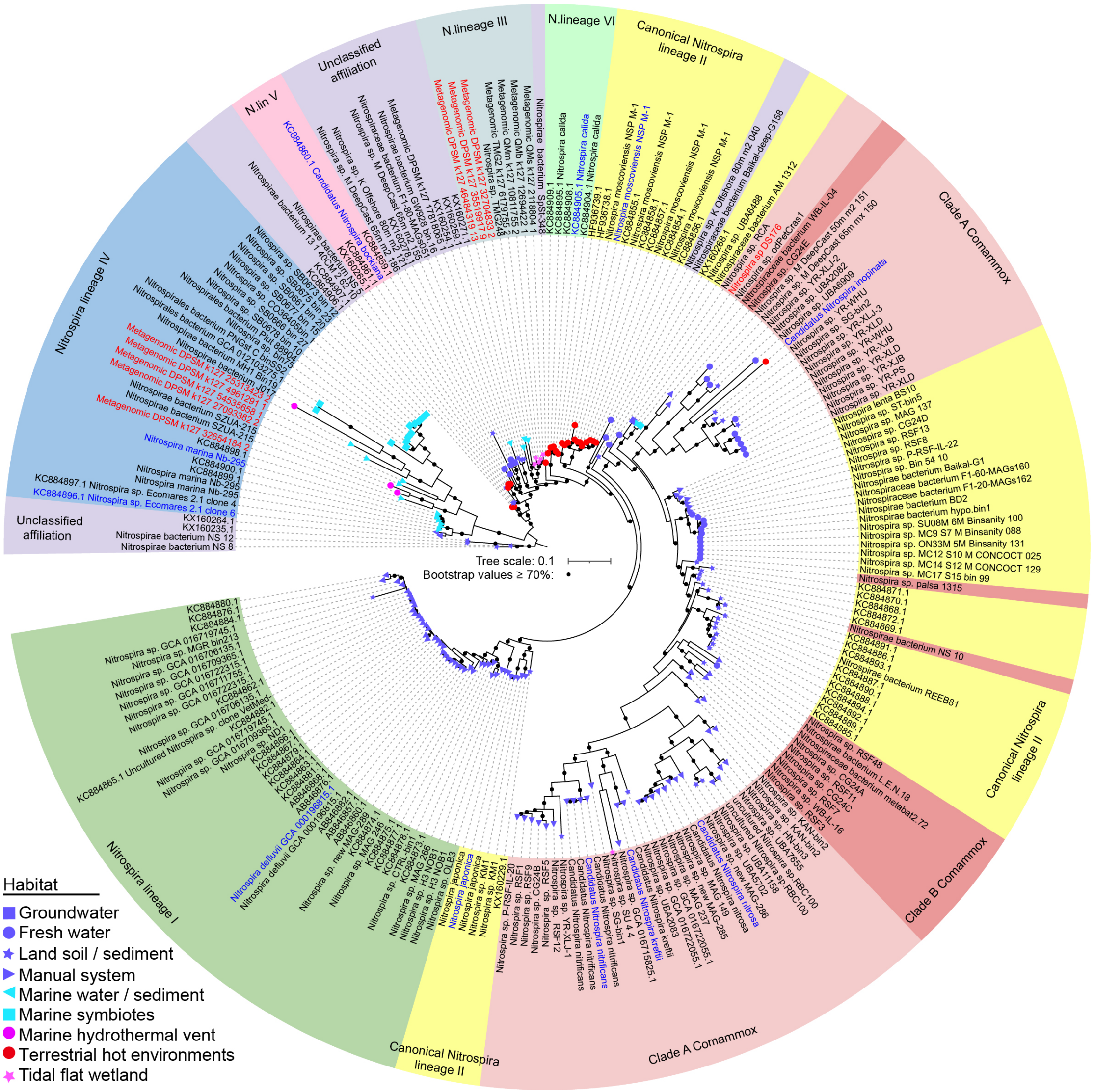

Fig. S4. Phylogenetic tree based on *nxrB* gene sequences from *Nitrospira*-like species. The maximum likelihood tree was constructed using 229 *nxrB* gene sequences (> 1 000 bp) from *Nitrospira*-like species with the GTR+F+R5 model and 1 000 ultrafast bootstrap replicates. Sequences derived from deep-sea metagenomes in this study were labeled in red, and those from cultured *Nitrospira* strains were labeled in blue. Branches with bootstrap values ≥ 70% are indicated with black circles.

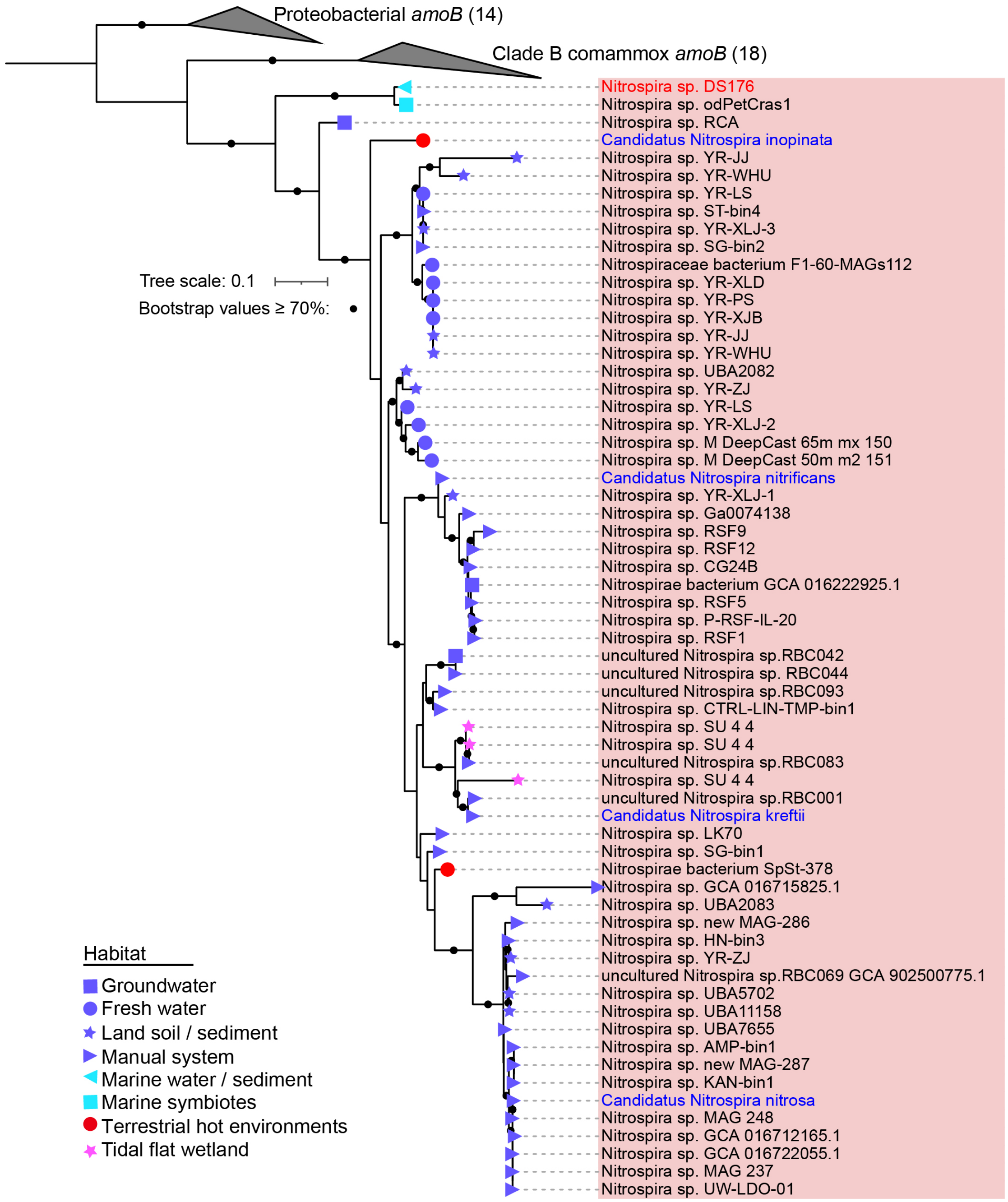

Fig. S5. Phylogenetic tree based on the *amoB* amino acid sequences. The maximum likelihood tree was constructed using 95 comammox and proteobacterial *amoB* amino acid sequences with the LG+R4 model and 1 000 ultrafast bootstrap replicates. Proteobacterial sequences are set as the outgroup. The number of sequences in each cluster is given in parentheses. Sequences derived from deep-sea metagenomes in this study were labeled in red, and those from cultured *Nitrospira* strains were labeled in blue. Branches with bootstrap values ≥ 70% are indicated with black circles.

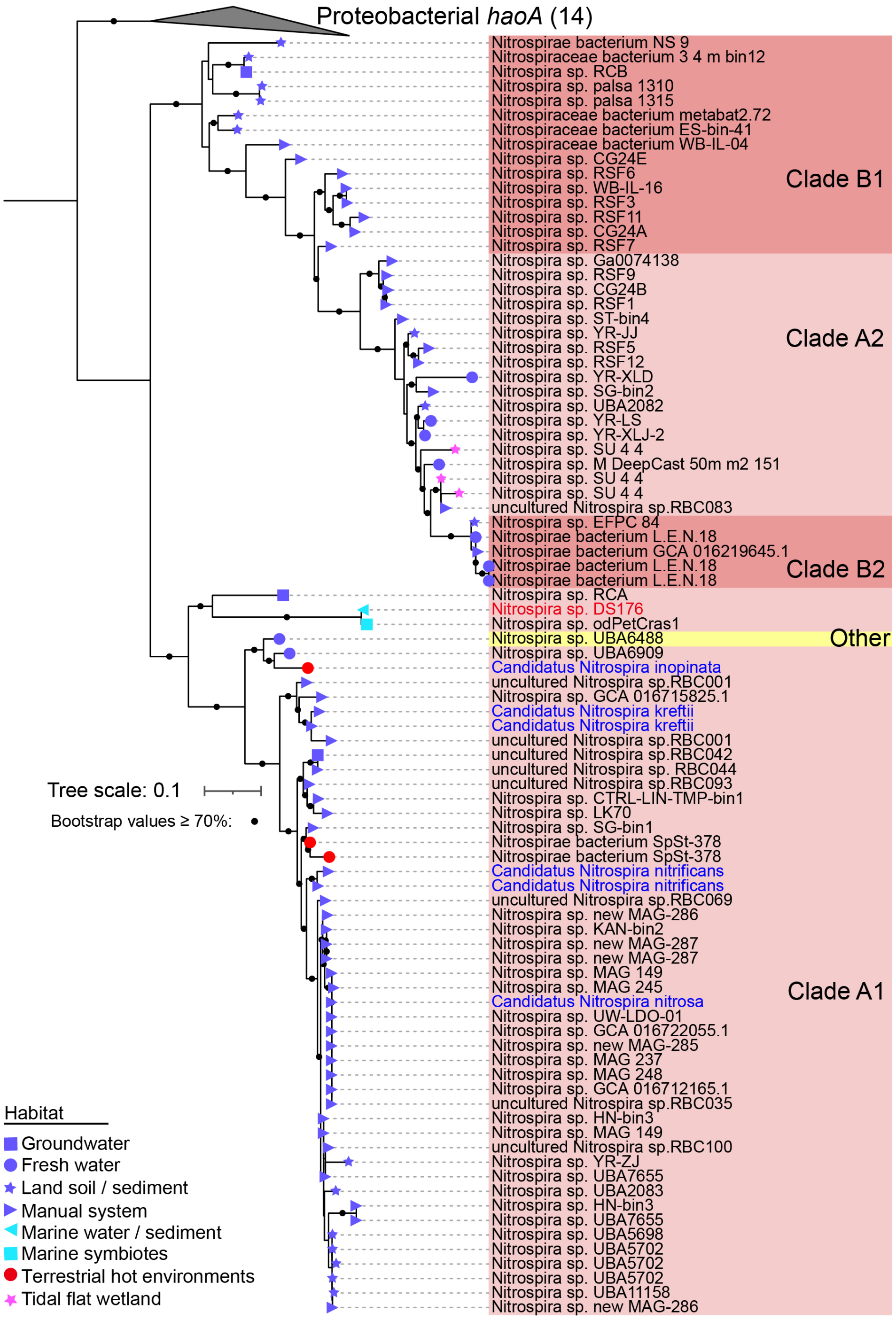

Fig. S6. Phylogenetic tree based on the *haoA* amino acid sequences. The maximum likelihood tree was constructed using 102 comammox and proteobacterial *haoA* amino acid sequences with the LG+R4 model and 1 000 ultrafast bootstrap replicates. Proteobacterial sequences are set as the outgroup. The number of sequences in each cluster is given in parentheses. Sequences derived from deep-sea metagenomes in this study were labeled in red, and those from cultured *Nitrospira* strains were labeled in blue. Branches with bootstrap values ≥ 70% are indicated with black circles.

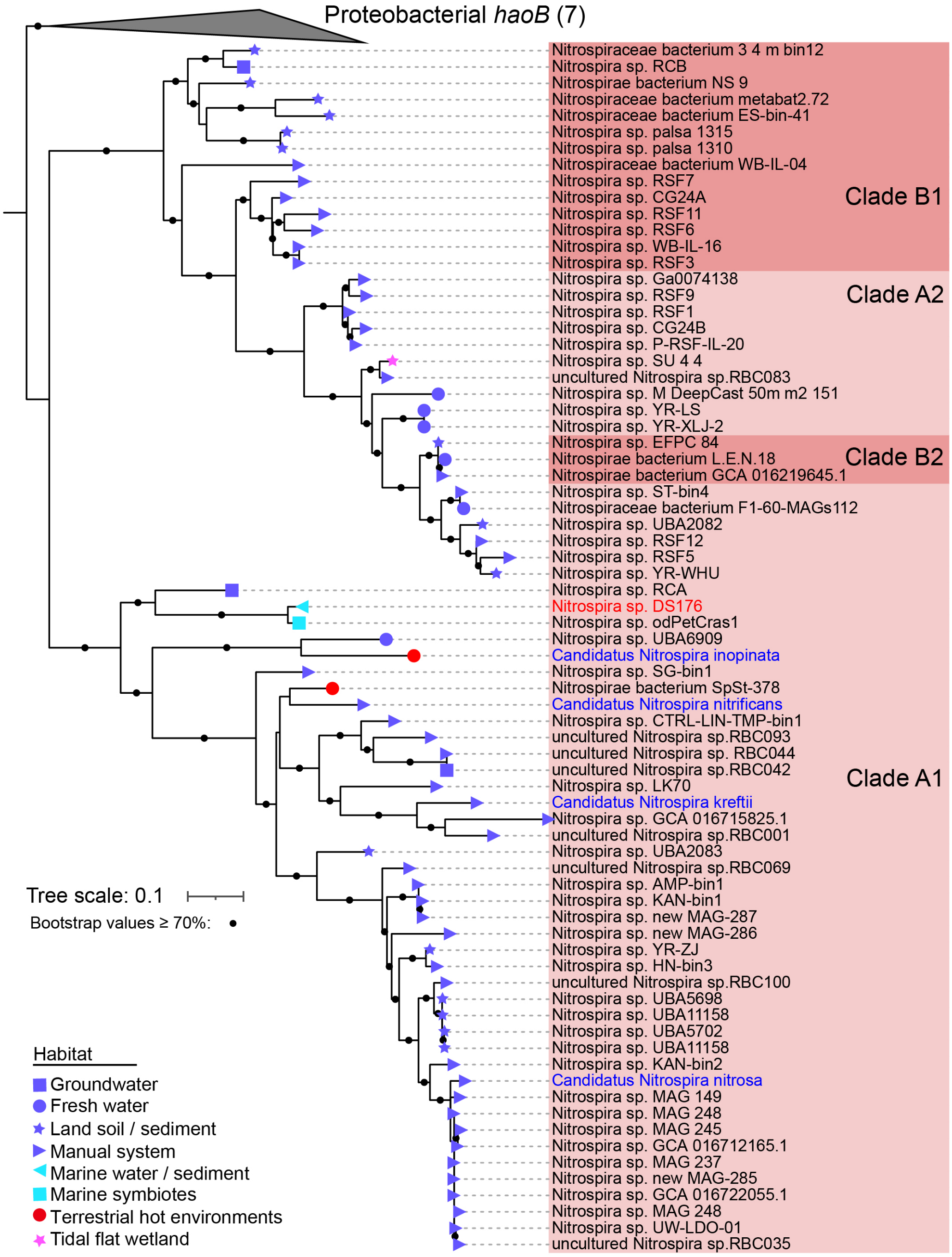

Fig. S7. Phylogenetic tree based on the *haoB* amino acid sequences. The maximum likelihood tree was constructed using 81 comammox and proteobacterial *haoB* amino acid sequences with the JTT+I+G4 model and 1 000 ultrafast bootstrap replicates. Proteobacterial sequences were set as the outgroup. The number of sequences in each cluster is given in parentheses. Sequences derived from deep-sea metagenomes in this study were labeled in red, and those from cultured *Nitrospira* strains were labeled in blue. Branches with bootstrap values ≥ 70% are indicated with black circles.

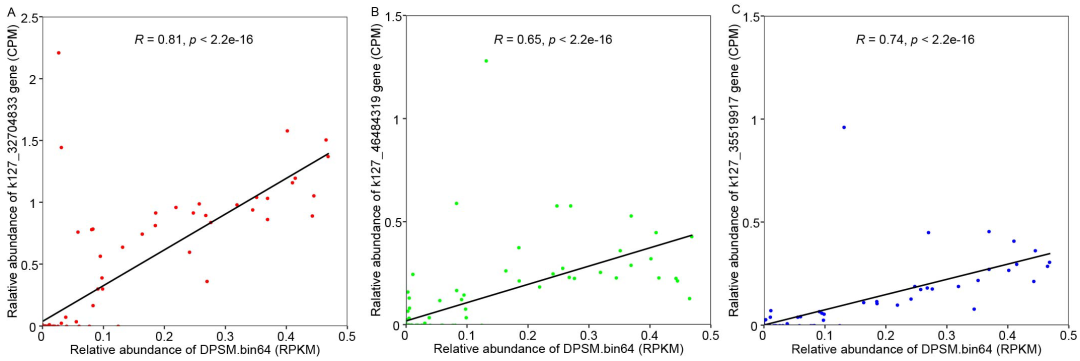

Fig. S8. The relationship between the relative abundances of MAG DS64 and those of different *nxrB* genes in marine metagenomes. The three full-length (1 290 bp) *nxrB* gene sequences retrieved from deep-seamount metagenomes are clustered with members of lineage III *Nitrospira*, as shown in Supplementary Fig. S4. Details regarding abundance information are provided in Supplementary Table S4.

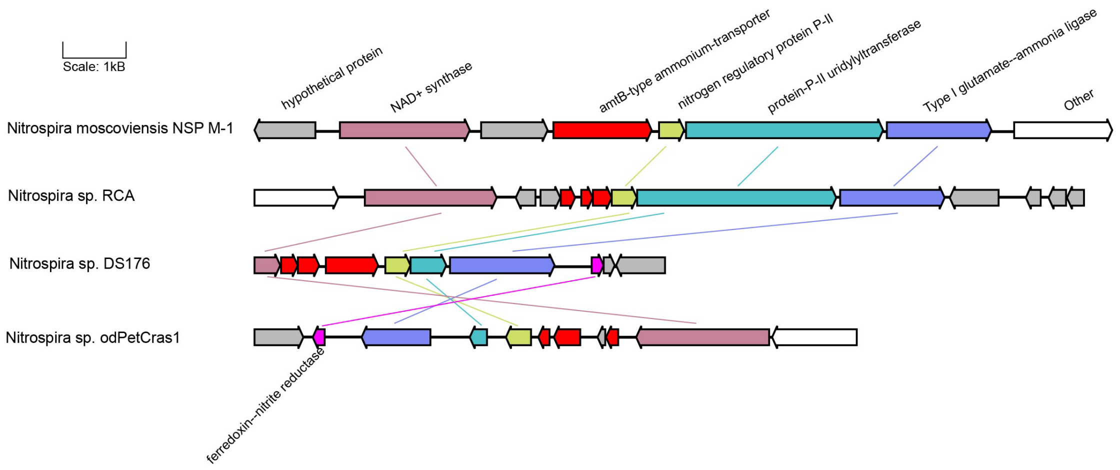

Fig. S9. Schematic representation showing *amtB* genes in four *Nitrospira* genomes. Arrows indicate genes and their transcriptional direction. Predicted protein functions are shown with colors and tags. Homologous genes were connected by lines and all genes were drawn to scale.

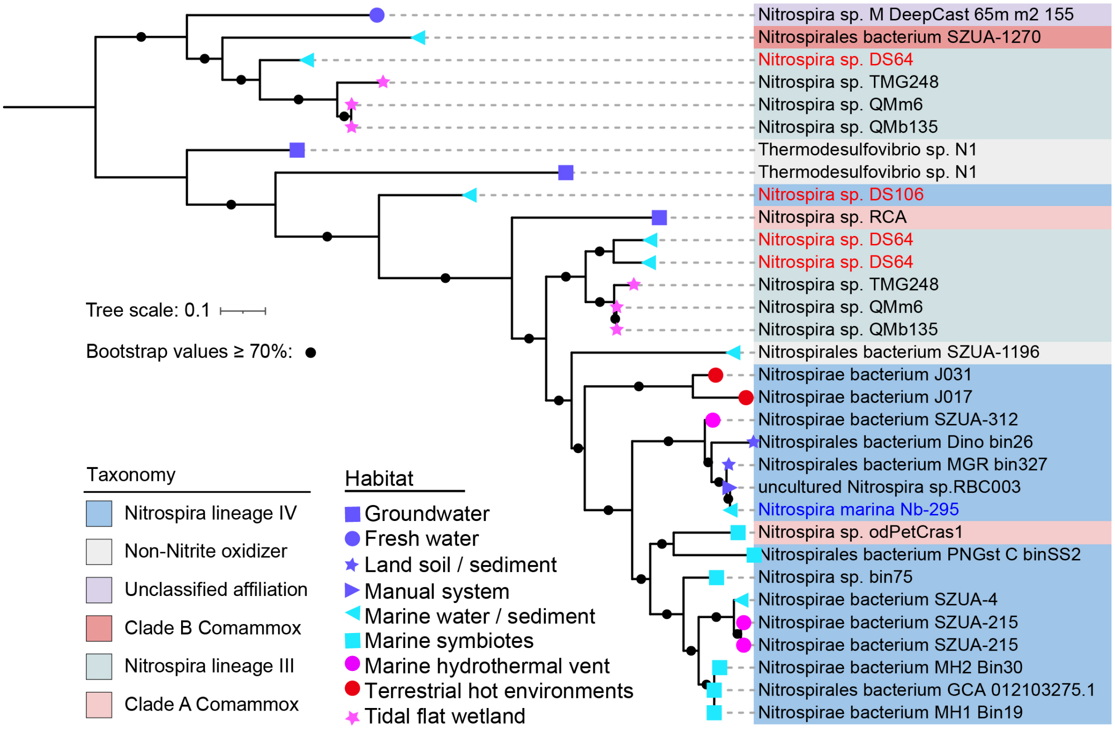

Fig. S10. Phylogenetic tree based on *mnhA* amino acid sequences. The maximum likelihood tree was constructed using 32 *mnhA* amino acid sequences with the LG+G4 model and 1 000 ultrafast bootstrap replicates. Sequences derived from deep-sea metagenomes in this study were labeled in red, and those from cultured *Nitrospira* strains were labeled in blue. Branches with bootstrap values ≥70% are indicated with black circles.

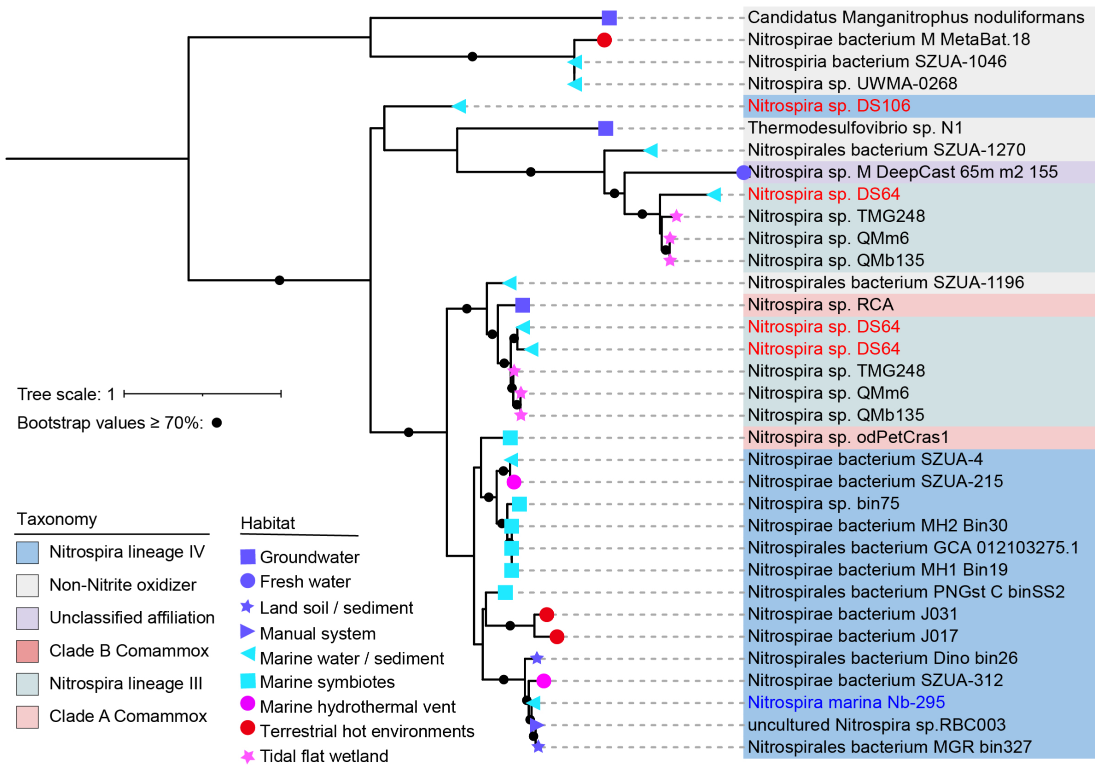

Fig. S11. Phylogenetic tree based on *mnhB* amino acid sequences. The maximum likelihood tree was inferred using 34 *mnhB* amino acid sequences from with the mtZOA+G4 model and 1 000 ultrafast bootstrap replicates. Sequences derived from deep-sea metagenomes in this study were labeled in red, and those from cultured *Nitrospira* strains were labeled in blue. Branches with bootstrap values ≥70% are indicated with black circles.

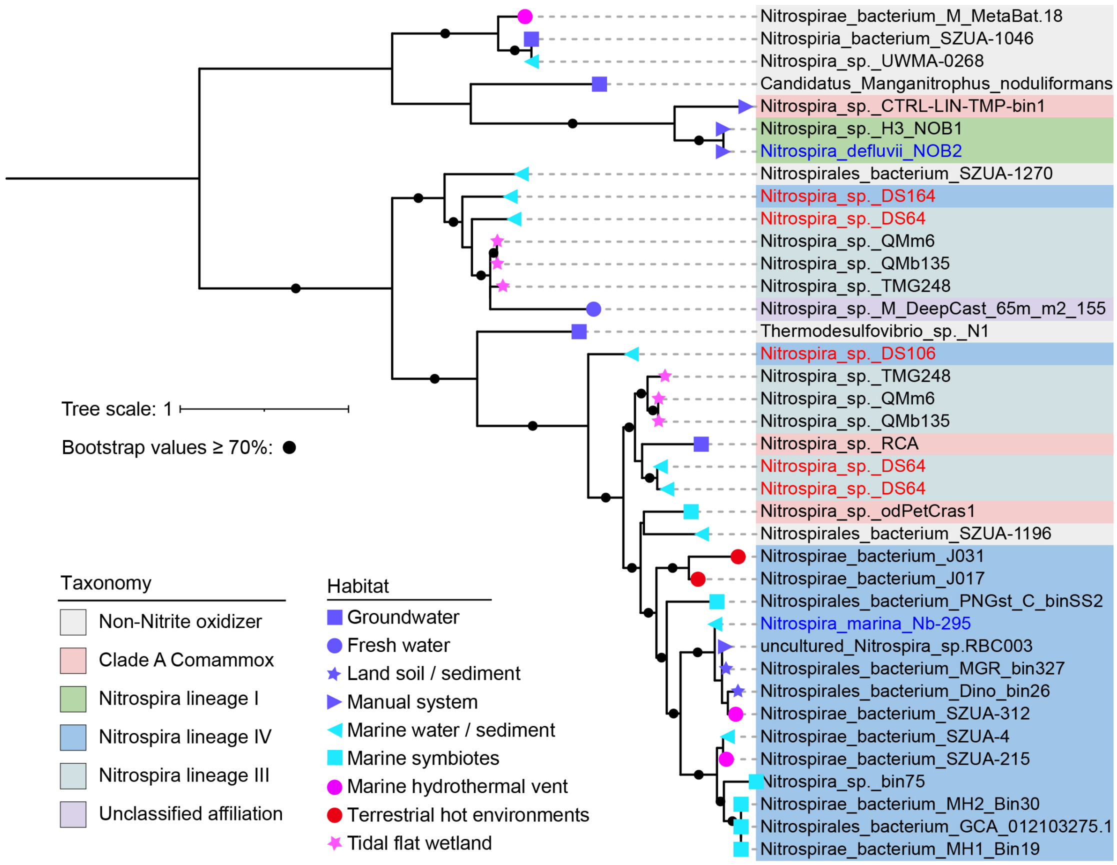

Fig. S12. Phylogenetic tree based on *mnhC* amino acid sequences. The maximum likelihood tree was constructed using 38 *mnhC* amino acid sequences from with the LG+G4 model and 1 000 ultrafast bootstrap replicates. Sequences derived from deep-sea metagenomes in this study were labeled in red, and those from cultured *Nitrospira* strains were labeled in blue. Branches with bootstrap values ≥70% are indicated with black circles.

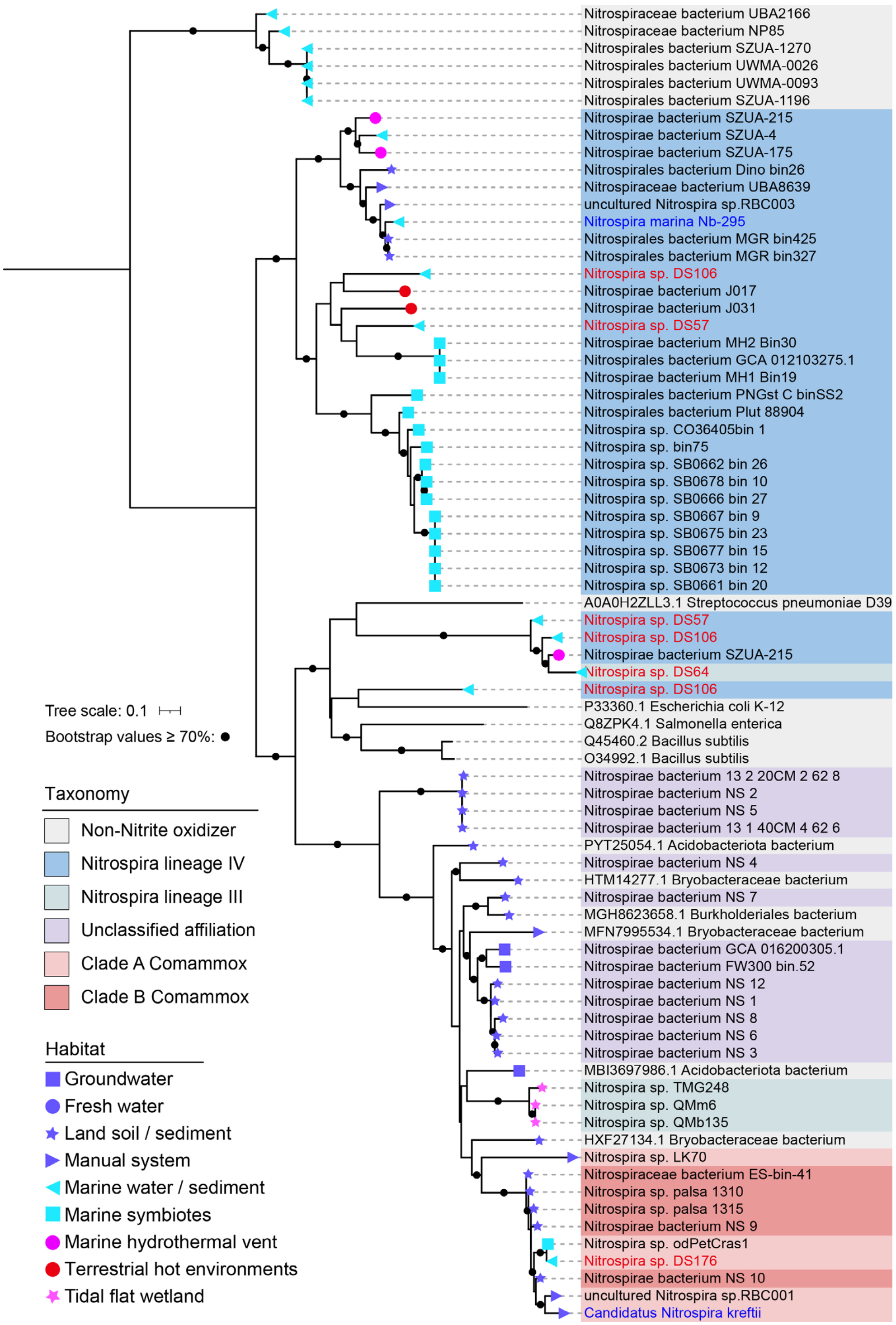

Fig. S13. Phylogenetic tree based on *opuA* amino acid sequences. The maximum likelihood tree was constructed using 65 *opuA* amino acid sequences with the LG+I+G4 model and 1 000 ultrafast bootstrap replicates. Sequences derived from deep-sea metagenomes in this study were labeled in red, and those from cultured *Nitrospira* strains were labeled in blue. Branches with bootstrap values ≥70% are indicated with black circles.

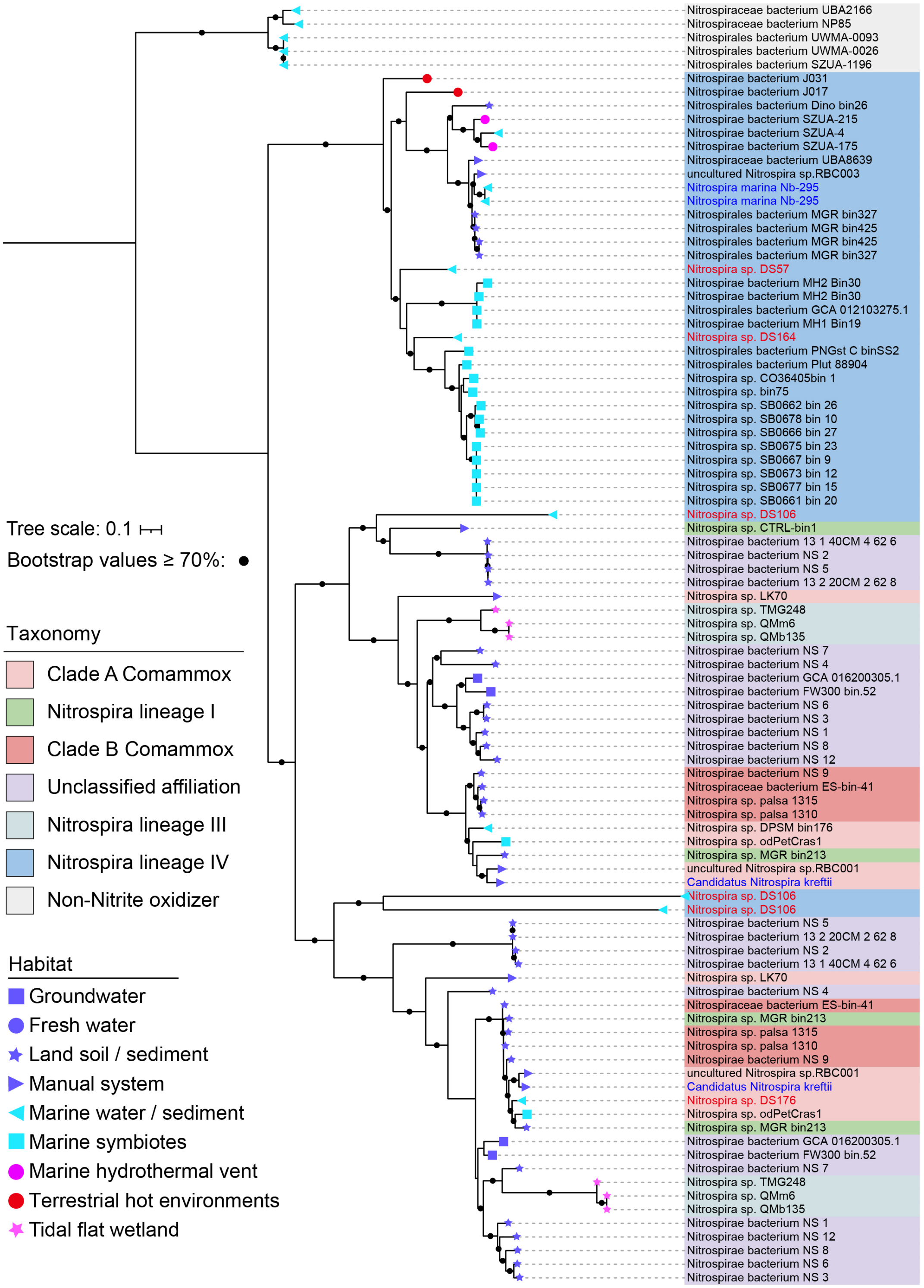

Fig. S14. Phylogenetic tree based on the *opuBC* amino acid sequences. The maximum likelihood tree was constructed using 94 *opuBC* amino acid sequences with the LG+F+G4 model and 1 000 ultrafast bootstrap replicates. Sequences derived from deep-sea metagenomes in this study were labeled in red, and those from cultured *Nitrospira* strains were labeled in blue. Branches with bootstrap values ≥70% are indicated with black circles.

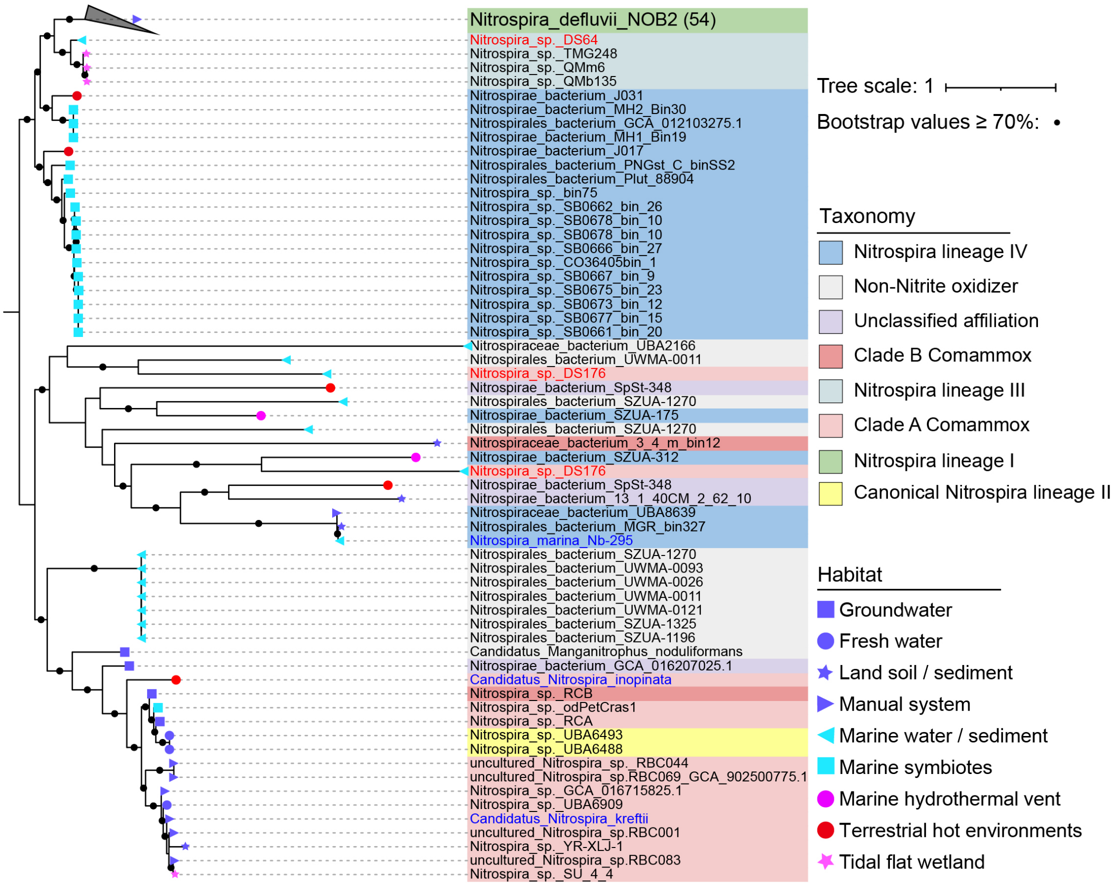

Fig. S15. Phylogenetic tree based on *upgA* amino acid sequences. The maximum likelihood tree was constructed using 115 *upgA* amino acid sequences with the LG+ G4 model and 1 000 ultrafast bootstrap replicates. Sequences derived from deep-sea metagenomes in this study were labeled in red, and those from cultured *Nitrospira* strains were labeled in blue. Branches with bootstrap values ≥70% are indicated with black circles.

Supplementary Tables

All supplementary tables are provided in an Excel table file.

Table S1. Properties of the sampling sites in this study.

Table S2. Genomic information of the *Nitrospirae* phylum.

Table S3. Average nucleotide identity (ANI) values from the genomic pairwise comparison.

Table S4. Relative abundance of MAG DS64 and lineage III *nxrB* genes in marine metagenomes.

Table S5. Relative abundance of MAG DS176 and comammox *amoAB* genes in marine metagenomes.

Table S6. Metabolic features of different *Nitrospira* lineages and sub-lineages genomes.

References

1. Hyatt D, Chen G-L, LoCascio PF, Land ML, Larimer FW, Hauser LJ. Prodigal: prokaryotic gene recognition and translation initiation site identification. *BMC Bioinf* 2010;**11**:119. https://doi.org/10.1186/1471-2105-11-119
2. Camacho C, Coulouris G, Avagyan V, Ma N, Papadopoulos J, Bealer K et al. BLAST+: architecture and applications. *BMC Bioinf* 2009;**10**:421. https://doi.org/10.1186/1471-2105-10-421
3. Pester M, Maixner F, Berry D, Rattei T, Koch H, Lucker S et al. *NxrB* encoding the beta subunit of nitrite oxidoreductase as functional and phylogenetic marker for nitrite-oxidizing *Nitrospira*. *Environ Microbiol* 2014;**16**:3055–71. https://doi.org/10.1111/1462-2920.12300
4. Yamada KD, Tomii K, Katoh K. Application of the MAFFT sequence alignment program to large data-reexamination of the usefulness of chained guide trees. *Bioinformatics* 2016;**32**:3246–51. https://doi.org/10.1093/bioinformatics/btw412
5. Criscuolo A, Gribaldo S. BMGE (Block Mapping and Gathering with Entropy): a new software for selection of phylogenetic informative regions from multiple sequence alignments. *BMC Evol Biol* 2010;**10**:210. https://doi.org/10.1186/1471-2148-10-210
6. Nguyen LT, Schmidt HA, von Haeseler A, Minh BQ. IQ-TREE: a fast and effective stochastic algorithm for estimating maximum-likelihood phylogenies. *Mol Biol Evol* 2015;**32**:268–74. https://doi.org/10.1093/molbev/msu300
7. Mistry J, Finn RD, Eddy SR, Bateman A, Punta M. Challenges in homology search: HMMER3 and convergent evolution of coiled-coil regions. *Nucleic Acids Res* 2013;**41**:e121. https://doi.org/10.1093/nar/gkt263
8. Bankevich A, Nurk S, Antipov D, Gurevich AA, Dvorkin M, Kulikov AS et al. SPAdes: a new genome assembly algorithm and its applications to single-cell sequencing. *J Comput Biol* 2012;**19**:455–77. https://doi.org/10.1089/cmb.2012.0021
9. Li C, Hu H-W, Chen Q-L, Chen D, He J-Z. Niche differentiation of clade A

comammox *Nitrospira* and canonical ammonia oxidizers in selected forest soils. *Soil Biology and Biochemistry* 2020;**149**:107925. https://doi.org/10.1016/j.soilbio.2020.107925
